# Dynamic induction of enantiomeric excess from a prochiral azobenzene dimer under circularly polarized light†
†Electronic supplementary information (ESI) available: Synthesis, ^1^H NMR spectra, ^13^C NMR spectra, UV absorption plots, HPLC traces, and CD spectra of the compounds; crystallographic information files. CCDC 1003754. For ESI and crystallographic data in CIF or other electronic format see DOI: 10.1039/c4sc01993h



**DOI:** 10.1039/c4sc01993h

**Published:** 2014-10-30

**Authors:** K. Rijeesh, P. K. Hashim, Shin-ichiro Noro, Nobuyuki Tamaoki

**Affiliations:** a Research Institute for Electronic Science , Hokkaido University , N20, W10, Kita-Ku , Sapporo 001-0020 , Hokkaido , Japan . Email: tamaoki@es.hokudai.ac.jp

## Abstract

The ability to photoinduce enantiomeric excess from the chirality of circularly polarized light (CPL) is pertinent to the study of the origin of homochirality in biomolecules. Such CPL-induced reactions, including both chirality generation and formation of partial enantiomeric imbalance, from nonchiral starting compounds have been known, however, only for the conversion of diarylolefins into chiral helicenes. In this study we synthesized three different prochiral molecules, each featuring a pair of photoisomerizable phenylazo moieties arranged symmetrically upon the phenyl rings of an sp^3^-hybridized carbon atom (**1**), the phenyl rings of [2.2]paracyclophane (**2**), and the *ortho* positions of a phenyl ring bearing a naphthyl unit (**3**), and then investigated the possibility of photoinducing enantiomeric excess under CPL. Irradiation of **1–3** with light induced *E* ↔ *Z* photoisomerizations of their azobenzene moieties, giving mixtures of their *EE*, *EZ*, and *ZZ* isomers in the photostationary state (PSS). Among these regioisomers, the *EZ* forms are chiral and existed as racemic mixtures of *R* and *S* stereoisomers. Upon CPL irradiation of **3**, circular dichroism (CD) revealed enantiomeric enrichment of one of the *EZ* stereoisomers; furthermore, irradiation with *r*- or *l*-CPL gave CD signals opposite in sign, but with equal intensity, in the PSS. In contrast, **1** and **2** did not give any detectable induced CD upon CPL irradiation. These experimental results can be explained by considering the different Kuhn anisotropy factors (*g*) of the (*R*)-*EZ* and (*S*)-*EZ* stereoisomers of **1–3**, assuming that the origin of the enantiomeric excess is the *enantio*-differentiating photoisomerization from *EZ* stereoisomers to nonchiral *EE* or *ZZ* regioisomers by *r*- or *l*-CPL. In short, we demonstrate the simultaneous induction of chirality and enantiomeric excess from a prochiral azobenzene dimer *via* a chiral regioisomer formed *in situ* upon CPL irradiation.

## Abstract 2

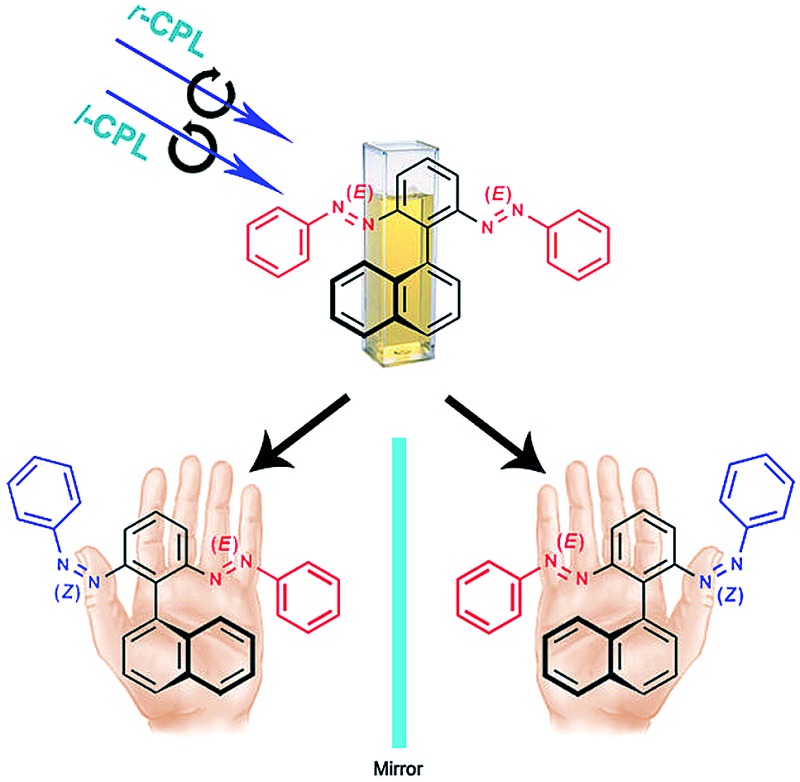
We demonstrate the simultaneous induction of chirality and enantiomeric excess from a prochiral azobenzene dimer *via* a chiral regioisomer formed *in situ* upon CPL irradiation.

## Introduction

Homochirality in Nature is one of the great unsolved and intriguing topics that has perplexed scientists, although many experiments and theories have been reported.^1–6^ Among the several physical sources that have been proposed^7^ to achieve enantiomeric imbalance in molecular systems, circularly polarized light (CPL) is the most studied chiral physical field,^7b,8–11^ considering the role of interstellar CPL^12^ in initial enantiomeric bias, and appears to be the strongest candidate for explaining the biomolecular homochirality that exists in Nature. Thus, enantiomeric enrichment of organic compounds through the action of CPL continues to be an area of high research interest.^13–15^

There are three CPL-induced reactions that can possibly enrich one enantiomer from a racemic pair: photodestruction, photoresolution, and absolute asymmetric synthesis.^7b,8,9^ The latter two are more important because some amount of enantiomeric excess remains in the reaction mixture after thorough photoirradiation. The main difference between photoresolution and absolute asymmetric synthesis is whether the chirality itself is generated during the photoreaction or not. In photoresolution, the starting compound is usually a racemic mixture of a chiral molecule, which can be converted reversibly to its mirror-image counterpart through a photochemical path. Such reversible *enantio*-differentiating photoisomerization of chiral molecules using CPL is known for some compounds based on the preferential interaction of *r*- or *l*-CPL with one of the enantiomers.^16–18^ A few groups have reported dynamic photoresolution occurring upon CPL irradiation of sterically overcrowded alkenes^19^ or bicyclic ketones;^20^ these species undergo photoresolution through a chiral discrimination pathway from the electronic ground states to a common excited state in which racemization of one of the enantiomers, excited selectively by *r*- or *l*-CPL, occurs. Recently, we reported the photoresolution of bicyclic^21^ and monocyclic^22^ azobenzene systems using CPL as a chiral source, where a ground state of the *cis* form was used as a common fast racemizing state to which the enantiomers of the *trans* form were selectively photoisomerized by *l*- or *r*-CPL. In contrast, only one example of absolute asymmetric synthesis with CPL has been reported: for nonchiral diarylolefins being photochemically converted to helicene derivatives with *enantio*-imbalance under CPL.^23^

In this paper we introduce a new CPL-induced reaction of a nonchiral compound forming a chiral product with an imbalance in the ratio of its enantiomers. In this concept, enantiomeric induction occurs from a prochiral azobenzene dimer through *in situ* formation of a chiral structure upon CPL irradiation at a suitable single wavelength. We propose a new absolute asymmetric synthesis, where enantiomeric imbalance is obtained as a result of an *enantio*-differentiating photoisomerization path from the photochemically formed enantiomers for one chiral regioisomer to a common ground state of its other non-chiral regioisomers.^24^ To the best of our knowledge, this example is the first demonstration of simultaneous induction of chirality and enantiomeric enrichment under CPL irradiation from a prochiral molecule through *enantio*-differentiating photoisomerization of a photochemically formed chiral structure.

## Experimental section

All solvents and chemicals were obtained from commercial sources and used without further purification, unless otherwise stated. NMR (^1^H and ^13^C) spectra were recorded using a JEOL ECX 400 spectrometer, with tetramethylsilane as the internal standard. Electrospray ionization (ESI^+^) mass spectrometry was performed using an AccuTOF instrument (JMS-T100LC; JEOL). Absorption spectra were recorded using an Agilent 8453 spectrophotometer. Circular dichroism (CD) spectra were recorded using a JASCO J-720 spectropolarimeter. Photoisomerization studies were conducted using radiation from an LED source of 365 nm and a super-high-pressure mercury lamp (500 W, Ushio) after passage through 436 nm filters. High-performance liquid chromatography (HPLC) was conducted on a Hitachi Elite La Chrome HPLC system using a Chiralpak IA column (Daicel Chemical Industries). Photostationary state (PSS) compositions were determined through HPLC analysis.

### Syntheses of **1** and **2**

See ref. 29 and 30.

### Synthesis of **3**

Nitrosobenzene (96.4 mg, 0.900 mmol) was added to a solution of **5** (53.0 mg, 0.230 mmol) and *t*-BuOK (101 mg, 0.900 mmol) in a mixture of DMSO (4 mL) and *t*-BuOH (1 mL) and then the reaction mixture was stirred for 12 h at room temperature. The resulting mixture was poured into saturated aqueous NH_4_Cl and extracted with CH_2_Cl_2_. The organic layer was washed with water and then dried (MgSO_4_). The solvent was evaporated and the residue subjected to column chromatography (SiO_2_; CH_2_Cl_2_/hexane, 4 : 6) to give an orange solid (20 mg, 21%). ^1^H NMR (400 MHz, CDCl_3_, 25 °C, TMS): *δ* 7.91–7.97 (m, 4H), 7.68 (t, *J* = 7.9 Hz, 1H), 7.54 (t, *J* = 7.7 Hz, 2H), 7.27–7.46 (m, 13H). ^13^C NMR (400 MHz, CDCl_3_, 25 °C, TMS): *δ* 152.79, 152.12, 139.54, 133.67, 133.61, 133.11, 131.09, 129.94, 129.02, 128.90, 128.49, 128.04, 127.94, 127.22, 126.16, 125.97, 125.42, 124.65, 123.15, 123.05, 120.81, 117.83; MS (ESI): calculated for C_28_H_20_N_4_, *m*/*z* 413.16 [M + H]^+^; found, 413.17.

### Single crystal X-ray analysis

A single crystal of **3** was mounted on a glass fiber. All measurements were made using a Rigaku R-Axis Rapid diffractometer and graphite-monochromated Mo-Kα radiation (*λ* = 0.71075 Å). Data were collected at a temperature of –100 °C to a maximum value of 2*θ* of 54.8°. An empirical absorption correction was applied, which resulted in transmission factors ranging from 0.653 to 0.977. The structure was solved using SIR2004 (ref. 25) and refined using SHELXL-97 with full-matrix least-squares techniques on *F* ^2^.^26^ Non-hydrogen atoms were refined anisotropically; hydrogen atoms were refined using the riding model. All calculations were performed using Crystal Structure 4.0 (Crystal Structure Analysis Package, Rigaku, Japan), except for refinement, which was performed using SHELXL-97.^26^ The image presented herein was generated using ORTEP-32 software.^27^

### CPL-induced CD

The CD instrument was purged with N_2_ for at least 20 min; the temperature was set to 25 °C prior to every measurement; spectra were measured between 390 and 625 nm with a standard sensitivity of 100 mdeg, a data pitch of 0.5 nm, a band width of 5 nm, a scanning speed of 20 nm min^–1^, and a response of 2 s, using a quartz cuvette (path length: 1 cm). Solutions were prepared in MeCN at a concentration of 10^–3^ M; reference CD data were collected in the same solvent. For CPL irradiation, the solution of **3** was kept in the cell holder of the CD spectrometer and irradiated directly with 436 nm light through film filters (TCPR or TCPL; MeCan Imaging, Japan) to produce *r*- or *l*-CPL (about 34 mW cm^–2^) for about 15 min, inducing positive and negative CD signals. The filters were alternated to produce *r*- or *l*- CPL to assess the reproducibility of the enrichment of the enantiomers. The induced spectra were smoothed using adjacent averaging method (*θ* values of 50 points in 25 nm were used to get the *θ* value at certain wavelengths). The smoothed spectra were adjusted to zero at 625 nm, assuming the *θ* value at 625 nm to be zero, and subtracted from the initial spectrum (CD spectrum prior to CPL irradiation) to obtain justified induced CD spectra. Under the same experimental conditions, compounds **1** and **2** did not provide any detectable CD signals after irradiation with *r*- or *l*-CPL. The intensity of the incident light in the CD spectrometer is lower than 0.16 mW cm^–2^ at any wavelengths during the measurement. Therefore the effect of the light during CD measurement on photoisomerization of the compound is negligible.

## Results and discussion

### Synthesis and structural characterization

**Fig. 1:**
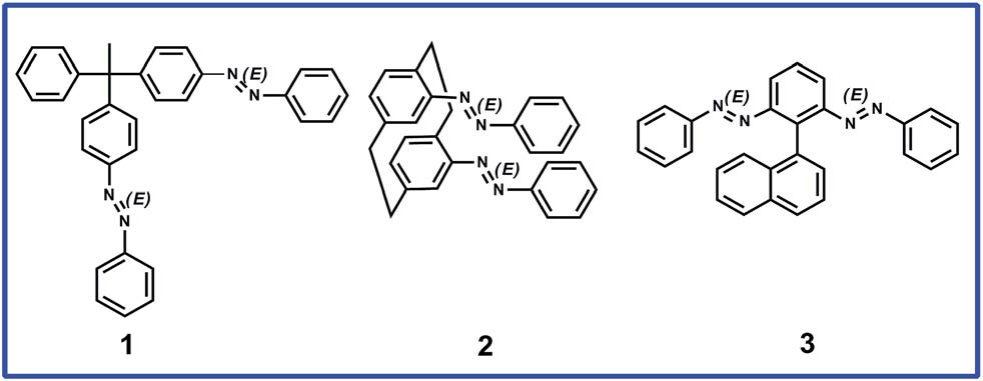
Three different types of prochiral azobenzene dimers.

In this study we designed molecules containing azobenzene units that can have *E* or *Z* regioisomeric structures between which transformation is possible by photochemical processes.^28^ We synthesized three prochiral molecules, **1**, **2**, and **3**, incorporating the azobenzene units as photoswitching components and point, plane, and axial chiral generating elements, respectively (Fig. 1). Because their phenylazo groups are connected symmetrically to their core frameworks (point, plane, or axis), these molecules are “non-chiral” when the two azobenzene units are in the *E* form; nevertheless, symmetry breaking would be possible through isomerization of a single azobenzene unit upon photo-irradiation. In compound **1**, each phenylazo group is linked to a benzene ring attached to a tetrahedral carbon atom bearing a methyl group and a phenyl group; in compound **2**, the phenylazo moieties are appended to the *p*-phenylene units of [2.2]paracyclophane. We recently reported the dynamic generation of point and planar chirality in compounds **1** (ref. 29) and **2**,^30^ respectively, upon conformational changes caused by *E*–*Z* photoisomerization of one of their azobenzene units. To apply our novel concept in an axially chiral system, we synthesized compound **3**, which features two phenylazo groups at the *ortho* positions of phenyl ring substituted by a naphthyl moiety.

The axial chirality of atropisomeric biaryl scaffolds is well discussed in the literature; the bulkiness of the dissymmetric substituents in the *ortho* positions plays a major role in the rotational stability to generate resolvable chiral structures (enantiomers).^31^ Thus, when designing compound **3**, we believed that the bulky phenylazo groups at the *ortho* positions of the benzene ring would be sufficient to block the rotation of the naphthyl unit around the central C–C bond, providing conformational stability to the atropisomers that would form upon photoirradiation.

**Scheme 1:**
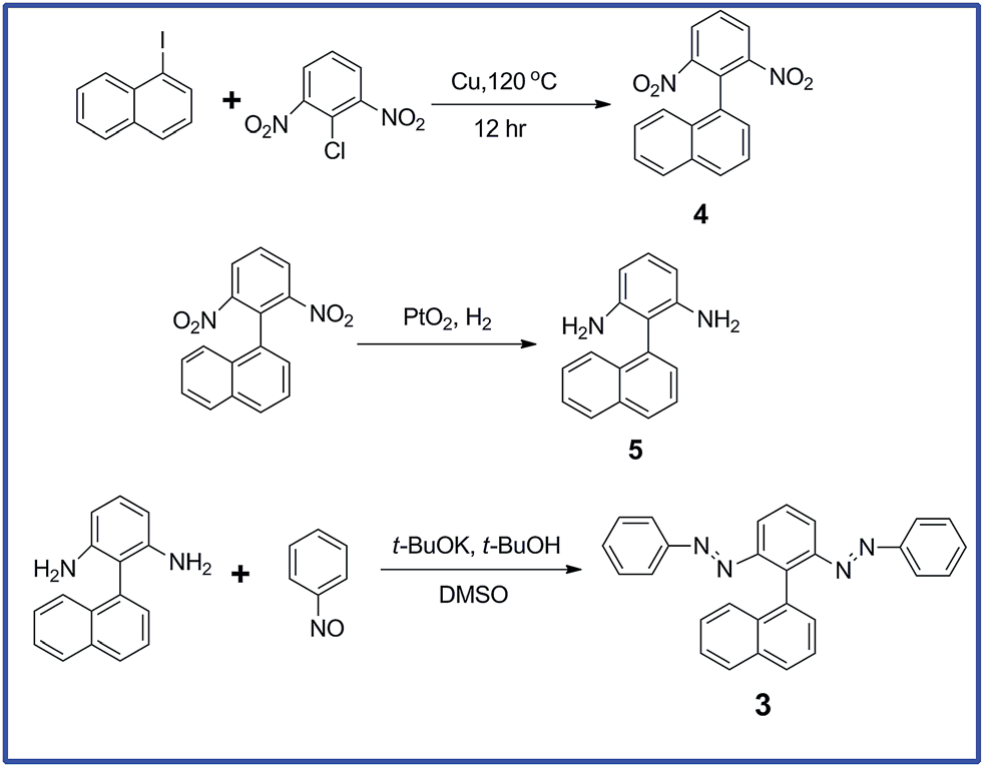
Synthesis of compound **3**.

We obtained each of these azobenzene dimers in moderate or low yield from corresponding diamines and nitrosobenzenes; the detailed synthetic processes for compounds **1** and **2** have been described previously.^29,30^ Scheme 1 outlines our synthesis of compound **3**. We obtained 1-(2,6-dinitrophenyl)naphthalene (**4**) through crossed Ullmann condensation between 1-iodonaphthalene and 1-chloro-2,6-dinitrobenzene in the presence of copper bronze.^32^ The PtO_2_/H_2_ reduction^33^ of **4** led to the corresponding diamine **5** in high yield, with product formation confirmed through NMR and mass spectral analyses. Although **5** has been reported as a byproduct from the action of hydrazines on β-naphthol in the presence of bisulphite,^34^ its efficient synthesis and characterization have not been discussed previously. We introduced the azo units through a simple base-catalyzed procedure, reacting **5** with nitrosobenzene in the presence of *t*-BuOK in *t*-BuOH/DMSO;^35^ we isolated a significant amount of the diazo compound **3**. The ^1^H NMR spectrum of **3** features the *ortho* protons of the azobenzene groups located upfield, appearing as a multiplet along with the naphthyl protons (*δ* 7.27–7.46); the other features of the ^1^H NMR spectrum matched were as predicted, with the structure supported by ^13^C NMR and mass spectral analyses (Fig. S1 and S2, ESI†).

**Fig. 2:**
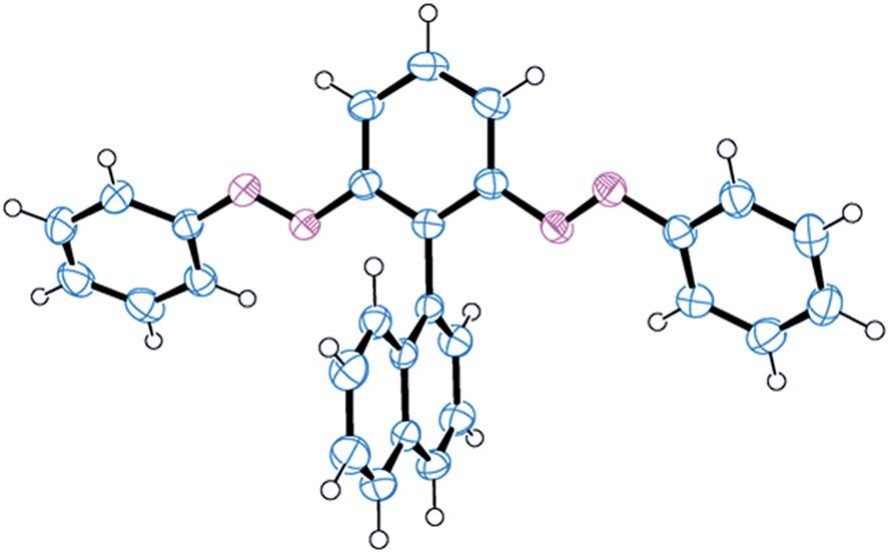
X-ray crystal structure of *EE*-**3**.

We confirmed the structure of **3** through single-crystal X-ray analysis of a crystal grown through slow evaporation of a CH_2_Cl_2_/hexane solution in the dark (Fig. 2). The crystal structure reveals that the azobenzene units in **3** existed in their thermodynamically stable *trans* form, with the phenyl and naphthyl moieties aligned perpendicularly, minimizing steric clashes between the bulky *ortho*-related phenylazo and naphthyl moieties. Moreover, the phenylazo groups were tilted slightly to the core benzene, presumably because of steric interactions with the naphthyl unit. These structural characteristics are comparable with those of atropisomeric *ortho*-substituted biphenyls, in which restricted rotation between two phenyl rings generates atropisomerism, potentially allowing isolation of the corresponding atropisomers (enantiomers) through chiral HPLC.^31^

### Photochemical isomerization and photoinduced chirality

**Fig. 3:**
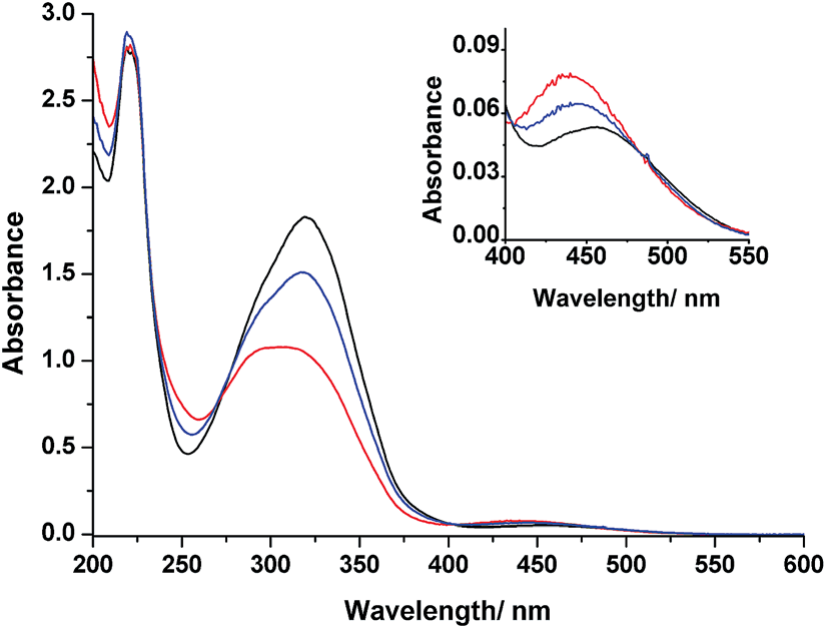
UV-Vis spectra of MeCN solution of **3** (4.69 × 10^–4^ mol L^–1^) at room temperature: (a) initial state prior to irradiation (black line); (b) PSS_366 nm_ (red line); (c) PSS_436 nm_ (blue line). Inset: enlarged view of the n–π* band.

Fig. 3 displays the absorption spectra of **3** in MeCN before and after irradiation with light at wavelengths of 366 and 436 nm at room temperature. The spectrum of **3** prior to irradiation features a band at 320 nm associated with π–π* transitions and a weak band at 453 nm corresponding to n–π* transitions. Upon UV irradiation at 366 nm, a gradual decrease in the π–π* band and concomitant increase in the n–π* band occurred, confirming photoisomerization from the *E* forms to the *Z* forms of the azobenzene groups. Further irradiation of the resulting solution at 436 nm induced a partial reversion to the original spectrum. These UV-Vis spectral changes are typical for azobenzenes.^36^ Because our design features two azobenzene units electronically decoupled through *meta* connection, we expected to observe independent *E*–*Z* isomerizations for each azobenzene unit—a situation supported by the appearance of isosbestic points at 273, 404, and 480 nm during the photochromic reactions of **3**. Thermal relaxation of the photoirradiated solution of **3** at 366 nm occurred very slowly (*k* = 3.15 × 10^–6^ s^–1^ at 30 °C), as observed from the changes in the absorption spectra in the dark (Fig. S20 and S21, ESI†). Moreover, **3** also exhibited reversible photoisomerizations between its *cis*- and *trans*-rich states (Fig. S5, ESI†) upon irradiation with visible light at wavelengths of greater than 500 nm (from the *trans*- to *cis*-rich state) and at 436 nm (from the *cis*- to *trans*-rich state), similar to the behavior of other *ortho*-substituted azobenzene derivatives reported recently.^37^

**Fig. 4:**
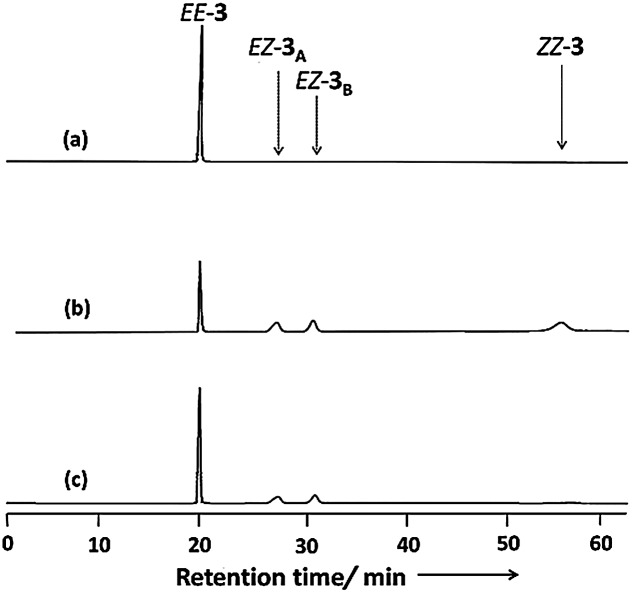
Chiral HPLC chromatograms (solvent: isopropanol/hexane, 1 : 4; flow rate: 1 mL min^–1^) of **3**: (a) before irradiation; (b) the PSS after irradiation at 366 nm; (c) the PSS after irradiation at 436 nm.

Fig. 4 displays chiral HPLC elution profiles of **3** before and after irradiation. Prior to irradiation, the chromatogram features a sharp single peak at a retention time (*R*_t_) of 20.26 min, suggesting that **3** existed initially in the *EE* form observed in the solid state. After irradiation with light at 366 nm, the chromatogram features three additional peaks at values of *R*_t_ of 28.24, 32.01, and 57.88 min, along with the initial signal for the *EE* form. Subsequent irradiation of the solution at 436 nm reverted the chromatogram characteristics, but left the second and third peaks at equal intensity after the irradiation process. *Z*-Azobenzene derivatives typically elute slowly in normal-phase HPLC (including chiral HPLC) because they are generally more polar than their corresponding *E* isomers.^21,38^ Accordingly, we suggest that the second, third, and fourth peaks represented the pair of enantiomers of *EZ*-**3** and the *ZZ*-**3** isomer, respectively.^39^

**Fig. 5:**
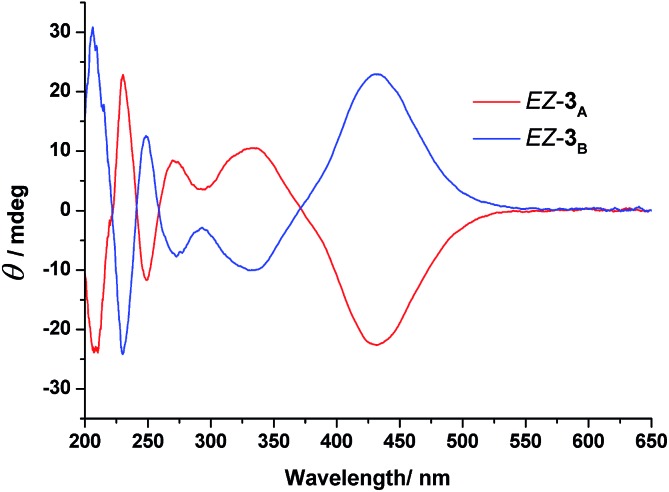
CD spectra of the enantiomers *EZ*-**3** in MeCN; (red line) *EZ*-**3_A_**, first eluted enantiomer; (blue line) *EZ*-**3_B_**, second eluted enantiomer. Concentration of each solution: 6.10 × 10^–4^ mol L^–1^.

To gain further information about the newly formed isomers after photoirradiation of *EE*-**3**, we isolated the second and third fractions of the HPLC chromatogram and measured their CD spectra. The CD spectrum of the second eluted fraction in MeCN featured three positive bands at 230, 275, and 335 nm and three negative bands at 210, 250, and 430 nm; a mirror-symmetrical Cotton curve appeared for the third fraction (Fig. 5). The UV-Vis absorption spectra of the fourth fraction resembled that of a *Z*-azobenzene derivative, with a characteristic π–π* transition band at 280 nm and an n–π* transition band at 436 nm (Fig. S11, ESI†).^36^ From these spectral data, we assign the second and third HPLC peaks to *EZ*-**3_A_** and *EZ*-**3_B_**, with one of the azobenzene units in the *trans* form and the other in the *cis* form, but with opposite conformations, and the fourth peak to *ZZ*-**3** with both azobenzene units in *cis* states.

To investigate the possibility of direct thermal racemization between *EZ*-**3_A_** and *EZ*-**3_B_**, we maintained *EZ*-**3_A_** in MeCN in the dark for one week at room temperature and used chiral HPLC to monitor the progress of its thermal back isomerization. Our HPLC analysis did not reveal a peak for *EZ*-**3_B_** during the thermal back isomerization from *EZ*-**3_A_** to *EE*-**3** (Fig. S12, ESI†), suggesting that the enantiomers are not thermally interconvertible. The HPLC chromatogram obtained after irradiating *EZ*-**3_B_** to PSS_436 nm_ was identical to that of the chromatogram obtained for the PSS obtained from *EE*-**3** at 436 nm (Fig. 4c). We found, however, that the HPLC elution profile of the photoreaction mixture of *EZ*-**3** before the PSS had been reached featured unequal peak areas for the *EZ* enantiomers in addition to the appearance of peaks for the *EE* and *ZZ* forms (Fig. S13, ESI†). These results suggest that the thermally stable *EZ*-**3_B_** enantiomer racemizes photochemically *via* the nonchiral *EE* or *ZZ* isomeric state.

Taking into consideration all of the data mentioned above, we propose a possible *EE* ↔ *ZZ* interconversion pathway through an intermediate chiral *EZ* isomeric state. Upon photoirradiation of the *EE* isomer, the independent isomerization of each azobenzene unit produces a mixture of *EE*, *EZ*, and *ZZ* regioisomers, which are photochemically interconvertible. Among these isomers, the *EZ* isomer formed through *E*–*Z* isomerization of a single azobenzene unit is chiral and exists as a racemic mixture of *R* and *S* stereoisomers. The (*R*)-*EZ* and (*S*)-*EZ* stereoisomers do not racemize thermally, but rather interconvert photochemically through the *EE* or *ZZ* isomeric state. The *ZZ* structure isomerizes either thermally or photochemically to the *EE* form *via* a mono-isomerized *EZ* state (Fig. S14 and S15, ESI†).

**Scheme 2:**
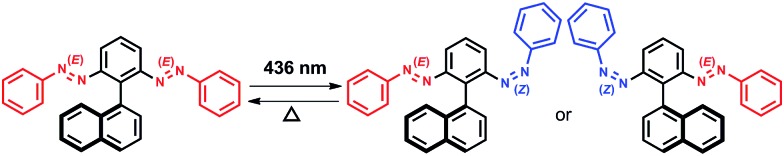
Chirality generation in **3** through *E*–*Z* photoisomerization.

Introduction of chirality to molecular entities usually involves unidirectional bond breaking or making steps, as seen in asymmetric synthesis. Herein, however, we demonstrate the dynamic generation of axial chirality in the azobenzene dimer **3** (Scheme 2), through *E*–*Z* photoisomerization of one of its azobenzene units upon photoirradiation; we also observed such behavior for **1** and **2**, which feature point and planar chirality, respectively.

### Photoresolution induced by CPL

The ability to induce absolute asymmetric synthesis through the application of CPL, where nonchiral compounds are converted to chiral products with an enantiomeric imbalance, is attractive because it provides a plausible mechanism for the origin of homochirality in bioorganic compounds.^16–22^ Our primary goal for this study was to investigate the feasibility of using the nonchiral compounds **1–3** as starting materials for reactions to form chiral products with enantiomeric excess through the photochemistry of CPL.

**Fig. 6:**
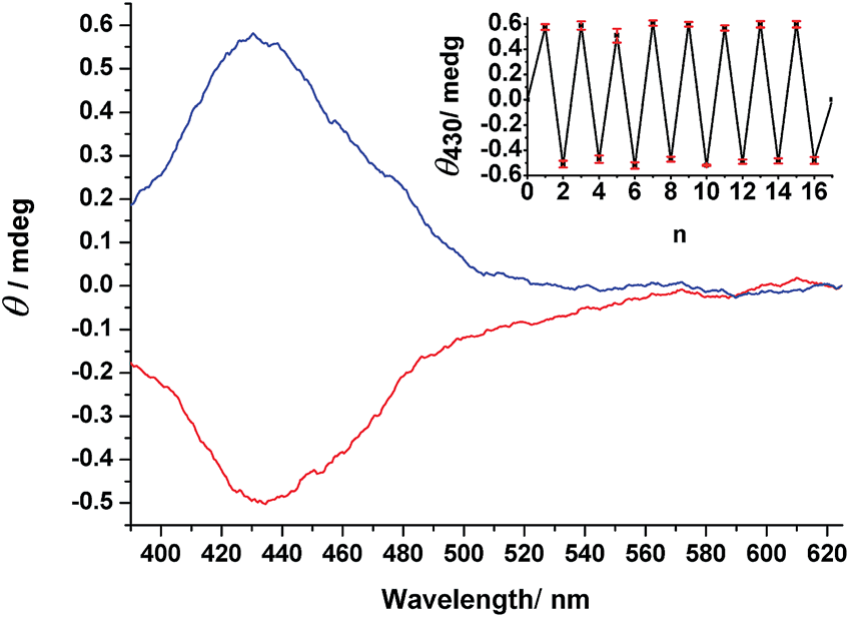
Induced CD spectra of an MeCN solution of **3** upon irradiation with *r*-CPL (blue line) and *l*-CPL (red line) at 436 nm. Inset: CD intensities at 430 nm for a solution of **3** upon alternating irradiation with *r*- and *l*-CPL (*n* = 1–16) and non-polarized light (*n* = 17), with a standard error of mean values from three independent experiments. Because the concentration was too high (1.33 × 10^–3^ M), it was impossible to measure the CD spectra at wavelengths of less than 390 nm.

We used *r*/*l*-CPL at 436 nm, the wavelength inducing the reasonable high concentration of *EZ* isomers at PSS and showing high Δ*ε* value for the enantiomers of *EZ*, to conduct photoresolution experiments of **1–3** under otherwise identical experimental conditions, monitoring these processes through CD spectroscopy. For **1** and **2**, we did not observe any induced CD spectra upon irradiation with either *r*- or *l*-CPL (Fig. S18 and S19, ESI†). Interestingly, irradiation of prochiral *EE*-**3** in MeCN with *r*-CPL at 436 nm resulted in a positive CD spectrum within 15 min. We confirmed this result through observation of the opposite CD signals, but with the same intensity, after irradiation of the same sample with *l*-CPL at 436 nm—and through comparison of the band shapes and positions in the induced spectra with those of the pure enantiomers of *EZ*-**3**. Successive irradiation of **3** with *r*- and *l*-CPL at the same wavelength led (Fig. 6) to CD spectral features with positive and negative signs, respectively, in the region of the n–π* transition band (390–625 nm). Further irradiation with nonpolarized light resulted in an inactive CD spectrum (*i.e.*, a photoracemized state was achieved). This process was reproducible over eight cycles without any deterioration of modulated signals (Fig. 6, inset), consistent with the CD measurements from the independent experiments.

We calculated the photoinduced enantiomeric excess (*ee*) using eqn (1) from the value of Δ*ε*_430_ (molar circular dichroism at 430 nm) of the pure enantiomer (11.6 L mol^–1^ cm^–1^) and the induced CD value (*θ*_430 nm_ = 0.5 mdeg) for a 1.33 × 10^–3^ M solution of **3** in MeCN at PSS_436 nm_, consisting of *EZ*-**3** as 24% (3.2 × 10^–4^ M) of the total isomers, assuming that the origin of the induced CD in *EZ*-**3** was some imbalance in the concentrations between the *R* and *S* stereoisomers at the photostationary state.

1 *ee*_PSS_ = {([*S*] – [*R*])/([*S*] + [*R*])} × 100 = 0.4%

where[*S*] – [*R*] = (induced *θ*_430_)/(32 980 × *Δε*_430_ × l cm)[*S*] + [*R*] = [*EZ*] at PSS_436 nm._

By substituting the experimentally obtained values of *ε*_436_ (1330 L mol^–1^ cm^–1^) and *Δε*_436_ (11.3 L mol^–1^ cm^–1^) obtained from the CD and UV absorption spectra of the pure enantiomers of *EZ*-**3** into eqn (2) (see ESI for derivation of the equation†), we calculated the theoretical *ee* to be 0.43% at PSS_436 nm_. The observed *ee* (*ee*_PSS_ = 0.4%) for the photoresolution of **3** upon irradiation with *r*- or *l*-CPL was in good agreement with the calculated value.

2 *ee*_PSS_ = Δ*ε*/2*ε* = *g*/2

Where *g* (anisotropy factor) = Δ*ε*/*ε*.^40^

We then measured the values of *ε*_436_ and *Δε*_436_ of *EZ*-**1** and *EZ*-**2** from the UV and CD spectra of the pure enantiomers (Fig. S9, S10, S16, and S17, ESI†). The expected *ee* calculated using eqn (2) from the values of *ε*_436_ (2336 L mol^–1^ cm^–1^) and *Δε*_436_ (0.15 L mol^–1^ cm^–1^) for *EZ*-**1** was too small (0.003% at 436 nm) to be detected by the CD instrument; the theoretical *ee* of *EZ*-**2** calculated from the values of *ε*_436_ (1907 L mol^–1^ cm^–1^) and *Δε*_436_ (4.2 L mol^–1^ cm^–1^) was 0.11%. Although the calculated *ee* for **2** was much higher than that for **1**, the *EZ* isomeric composition at PSS_436 nm_ was much lower (12%), making that system impractical for the detection of photochemical deracemization under CPL. Thus, compound **3** was the most suitable azobenzene dimer for studying CPL-induced reactions to enrich one of the enantiomers, owing to its larger value of *g* (8.5 × 10^–3^) and a higher [*EZ*] ratio (24%) at PSS_436 nm_ after CPL irradiation, relative to those of **1** and **2**.

**Scheme 3:**
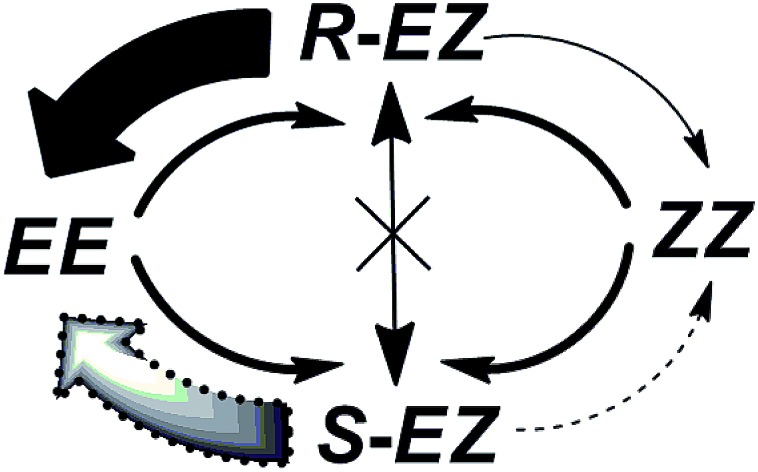
*Enantio*-differentiating photoisomerization pathways for *EZ* isomer formation during irradiation with one of CPLs. Solid arrows represent much higher reaction rate of the photoisomerization paths than the corresponding dotted arrows.

As stated earlier, the *EE*, *EZ*, and *ZZ* isomers are photochemically interconvertible; they establish an equilibrium composition upon photoirradiation. Both the *EE* and *ZZ* isomers are achiral, but the *EZ* isomer is chiral and exists as a mixture of *R* and *S* stereoisomers. The reversible photoisomerizations from the *R* and *S* enantiomers of *EZ* to the nonchiral *EE* or *ZZ* state occur with same efficiency when irradiating with nonpolarized light. Under *r*- or *l*-CPL irradiation, however, the *R* and *S* enantiomers of the *EZ* form photoisomerize selectively (solid and dotted arrows in Scheme 3) to nonchiral *EE* or *ZZ* ground states. This repeated *EZ* state [(*R*)-*EZ* or (*S*)-*EZ*] to *EE* or *ZZ enantio*-discriminating photoisomerization pathway, and the reverse nonenantio-discriminating photoisomerization from *EE* or *ZZ* state to the *EZ* isomer, upon CPL irradiation leads to an enantiomeric imbalance in the system at the PSS. As a result, a chiral product with a partial *enantio*-imbalance formed from a nonchiral compound.

Only one example of absolute asymmetric synthesis has ever been reported when using CPL as the physical chiral origin.^23^ In that reaction, *trans*-diarylolefins with different aromatic rings photoisomerized to *cis*-diarylolefins, which existed as mixtures of thermally interconvertible helical enantiomers. Upon further photoabsorption, the *cis*-diarylolefins underwent cyclizations to form dihydrohelicenes, which were chemically converted to helicene through oxidation.

If absolute asymmetric synthesis is defined as the reaction of an achiral compound to give a chiral product without any sources of chemical chirality, our present CPL-induced reaction for **3** to form a chiral product with *enantio*-imbalance is seemingly a new absolute asymmetric synthesis using CPL. Actually it is a simultaneous photoresolution process of a photochemically formed racemic mixture. To the best of our knowledge, this paper is the first to demonstrate the generation of chirality through *in situ* formation of asymmetry and photoresolution in a single molecule.

## Conclusions

We have synthesized a new class of azobenzene dimers featuring two symmetrically arranged azobenzene units, in which a photoinduced conformational change of one of the azobenzene moieties can be used to generate asymmetry in the molecules, thereby allowing an investigation of CPL-induced photoresolution. We have demonstrated that, upon photoirradiation, *E*–*Z* photoisomerization of one of the azobenzene units connected to an sp^3^-hybridized carbon atom in **1** or to the phenyl rings of the [2.2]paracyclophane core in **2** generates central and planar chirality, respectively. We also present the novel structure **3**, with two azobenzene units substituted at the *ortho* positions of a phenyl ring presenting a naphthyl unit, that induces axial chirality upon irradiation with light of suitable wavelengths. Most interestingly, we applied **3** in studies of reversible *enantio*-differentiating photoisomerizations induced by *r*- and *l*-CPL at 436 nm. For the first time, we have demonstrated the simultaneous generation of chirality and *ee* from a nonchiral structure upon CPL irradiation at a single wavelength, and propose a new mechanism for CPL-induced photoresolution from the ground states of the *R* and *S* enantiomers of the *EZ* form to a common ground state of nonchiral *EE* or *ZZ* forms. We believe that such a chiral-to-nonchiral *enantio*-differentiating photoisomerization pathway upon irradiation with *r*- or *l*-CPL constitutes a molecular model, involving a partial photoresolution mechanism, that might explain the chiral imbalance found in Nature. We also anticipate that *in situ* generation of chirality from prochirality through the action of light, without any complex chemical reactions, will be a promising method for operating optical memory devices; such studies are currently ongoing.
