# Supplementary material for: Dynamic induction of enantiomeric excess from a prochiral azobenzene dimer under circularly polarized light
Source: Chem Sci. 2014 Oct 30;6(2):973–80. doi: 10.1039/c4sc01993h (PMC5811134; doi:10.1039/c4sc01993h)
Supplement: Supplementary file 1 [file SC-006-C4SC01993H-s001.pdf]

Electronic Supplementary Material (ESI) for Chemical Science.
This journal is © The Royal Society of Chemistry 2014

Supporting Information

Dynamic Induction of Enantiomeric Excess from a Prochiral Azobenzene Dimer Under Circularly Polarized Light

K. Rijeesh, P.K. Hashim, Shin-ichiro Noro, and Nobuyuki Tamaoki*

Research Institute for Electronic Science, Hokkaido University, N20, W10, Sapporo, Hokkaido, 001-0020, Japan

Contents:

1. General experimental methods……………………………………………………S2
2. Synthesis…………………………………………………………………………..S3
3. NMR spectra……………………………………………………………………S4
4. Absorption spectra……………………………………………………..….S6
5. HPLC chromatogram……………………………………………………S10
6. CD spectra…………………………………………………………….…….S12
7. Kinetic studies……………………………………………………………….S14
8. Estimation of the enantiomeric excess after CPL irradiation…………………..S15
9. Crystal data………………………………………………………………..S16

1. General experimental methods.

All solvents and chemicals were obtained from commercial sources and used without further purification, unless otherwise stated. ^{1}H and ^{13}C NMR spectra were recorded using a JEOL ECX 400 spectrometer, with tetramethylsilane as the internal standard. Electrospray ionization (ESI^{+}) mass spectrometry was performed using an AccuTOF instrument (JMS-T100LC; JEOL). X-ray crystallographic data were acquired using a Bruker Smart Apex diffractometer. Absorption spectra were recorded using an Agilent 8453 spectrophotometer. CD spectra were recorded using a JASCO J-720 spectrophotometer; baseline correction and binomial smoothing were applied to the spectra. Photoisomerization studies were conducted using radiation from an LED source of 365 nm and a super-high-pressure mercury lamp (500 W, Ushio) after passage through 436-nm filters. High-performance liquid chromatography (HPLC) was performed using a Hitachi Elite La Chrome HPLC system and a Chiralpak IA column (Daicel Chemical Industries). Compositions of photostationary states were determined through HPLC analysis. A mixture of isopropanol and hexane (1:4) was used as the eluent in the HPLC experiments.

2. Synthesis

Compound 4: A mixture of 1-chloro-2,6-dintrobenzene (250 mg, 1.23 mmol), iodonaphthalene (320 mg, 1.26 mmol), and copper bronze (320 mg, 5.04 mmol) was heated at 120 °C for 12 h (until the spot for dintrochlorobenzene disappeared in TLC analysis). The product was extracted into CH_2Cl_2; the combined extracts were washed with water and dried ($MgSO_4$). The solvent was evaporated under vacuum and the residue subjected to column chromatography (SiO_2; CH_2Cl_2/hexane, 2:3) to give a pale yellow solid (38% yield). ^{1}H NMR (400 MHz, $CDCl_3$, 25 °C, TMS); δ = 8.16 (d, *J* =8.2 Hz, 2H), 7.92 (dd, *J* =8.2, 8.3, 2H), 7.81 (t, *J* =8.2 Hz, 1H), 7.49–7.53 (m, 2H), 7.42–7.46 (m, 1 H), 7.33–7.34 (m, 2H).

Compound 5: Compound **4** (100 mg, 0.34 mmol) was dissolved in a 2:1 mixture of EtOH and 1,4-dioxane (5 mL) and then the reaction flask was evacuated and backfilled with Ar three times. PtO_2 (10 mg, 0.4 mmol) was added under an Ar atmosphere and then the atmosphere was changed from Ar to H_2. The reaction mixture was stirred at room temperature until TLC (mobile phase: 20% EtOAc/hexane) revealed a single spot, approximately overnight. The catalyst was removed by filtration over Celite; the solvent was evaporated to dryness under vacuum to yield a brownish solid (71 mg, 90% yield). ^{1}H NMR (400 MHz, $CDCl_3$, 25 °C, TMS): δ = 7.90–7.92 (m, 2H), 7.67 (d, *J* =8.5 Hz, 1H), 7.58–7.61 (m, 1H), 7.49–7.54 (m, 2H), 7.42–7.46 (m, 1H), 7.07 (t, *J* =7.9 Hz, 1H), 3.32 (s, 4H); MS (ESI$^+$): calcd for $C_{16}H_{14}N_2$ $[M + H]^+$: *m/z* 235.12; found: 235.11.

3. NMR spectra

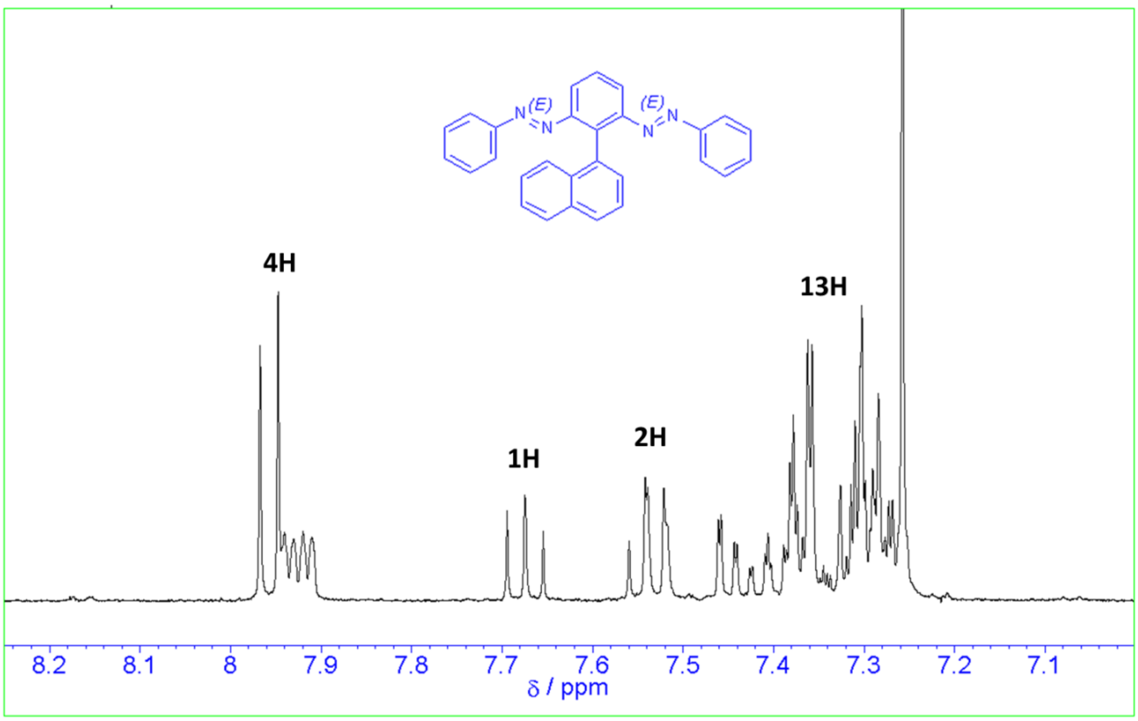

Figure S1. ^{1}H NMR spectrum of compound **3** in $CDCl_3$ at room temperature.

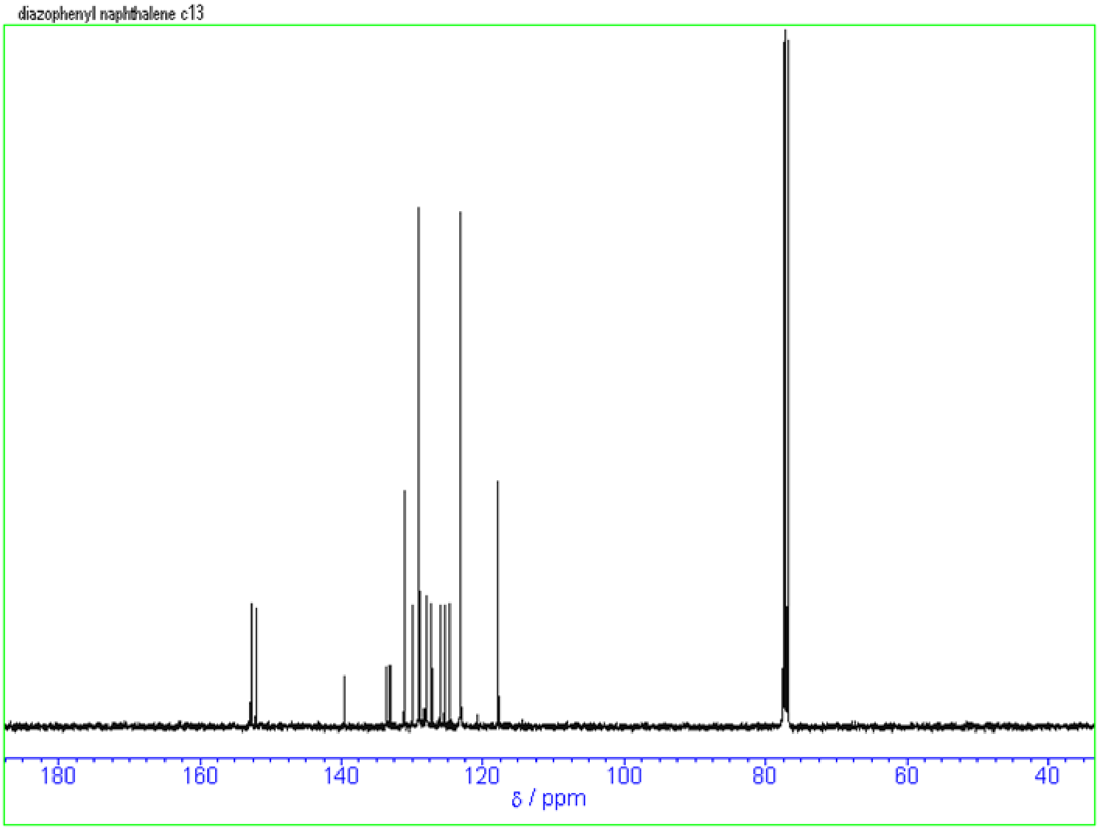

Figure S2. ^{13}C NMR spectrum of compound **3** in $CDCl_3$ at room temperature.

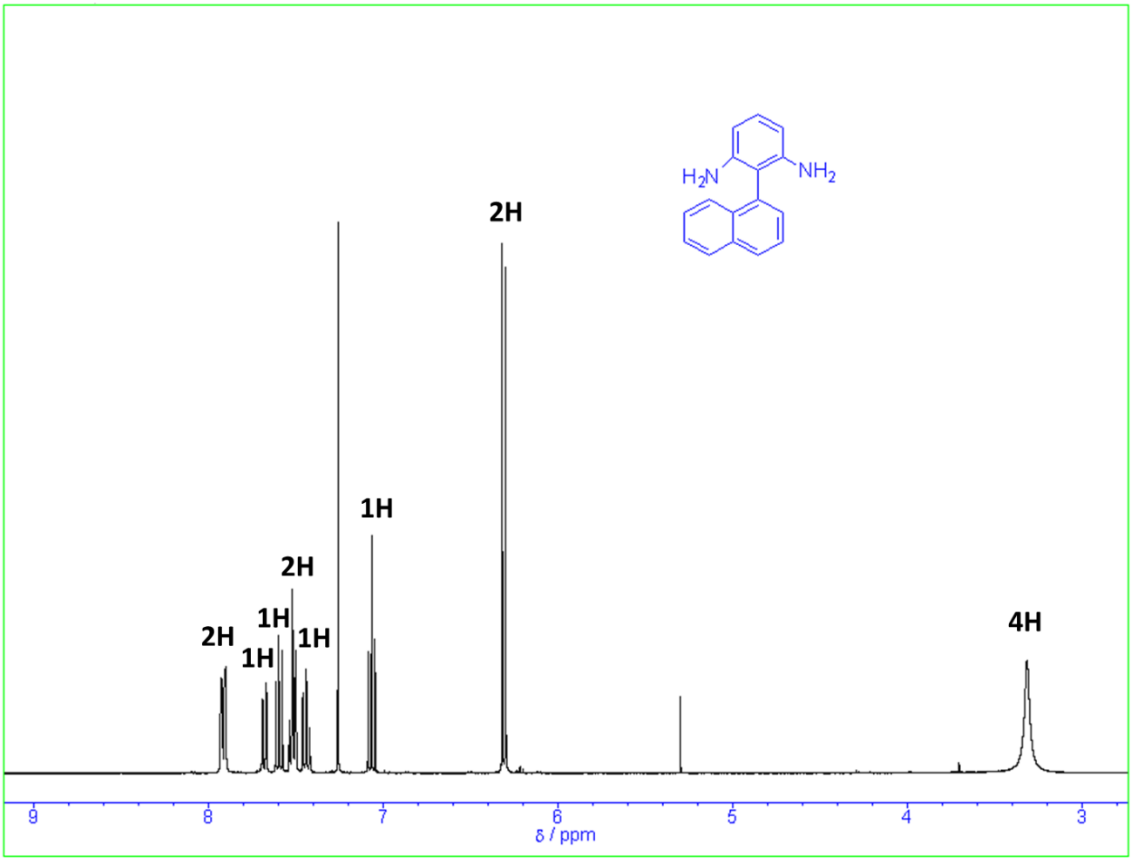

Figure S3. ^{1}H NMR spectrum of compound **5** in $CDCl_3$ at room temperature

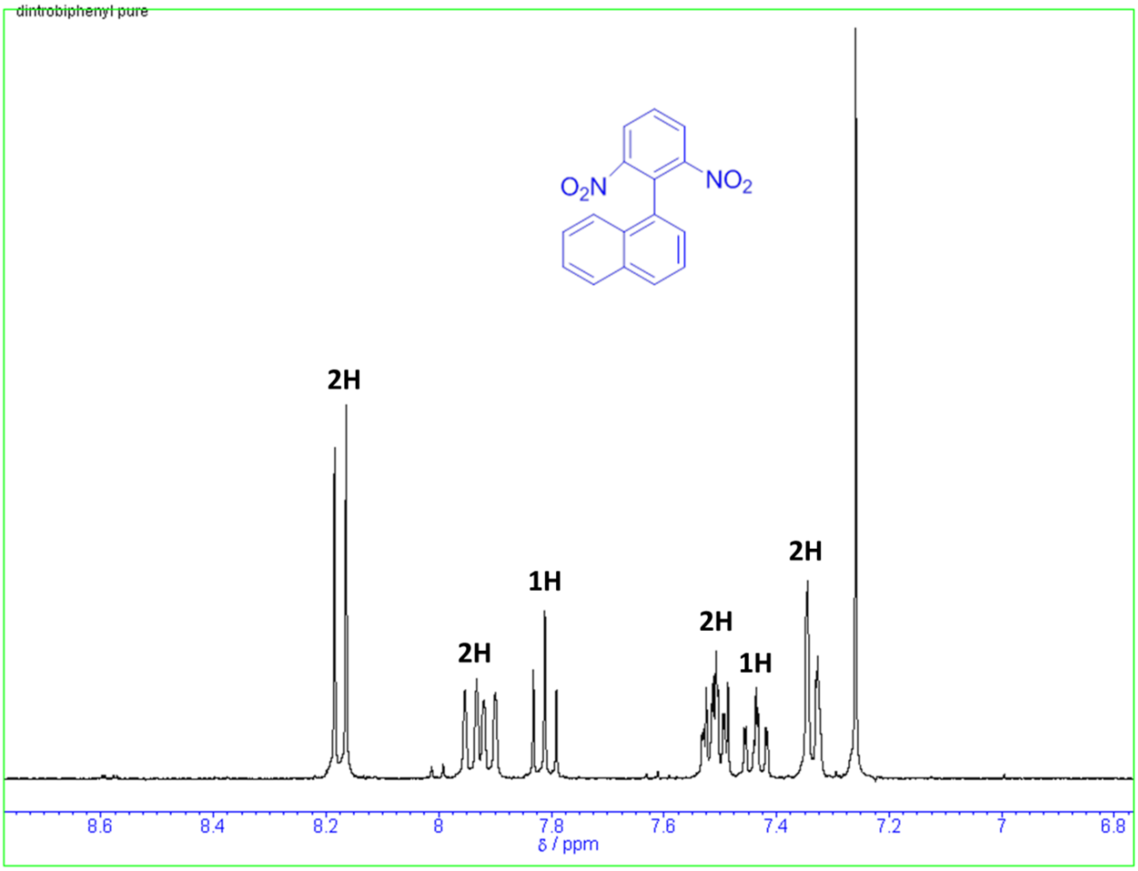

Figure S4. ^{1}H NMR spectrum of compound **4** in $CDCl_3$ at room temperature.

4. UV–Vis absorption spectra

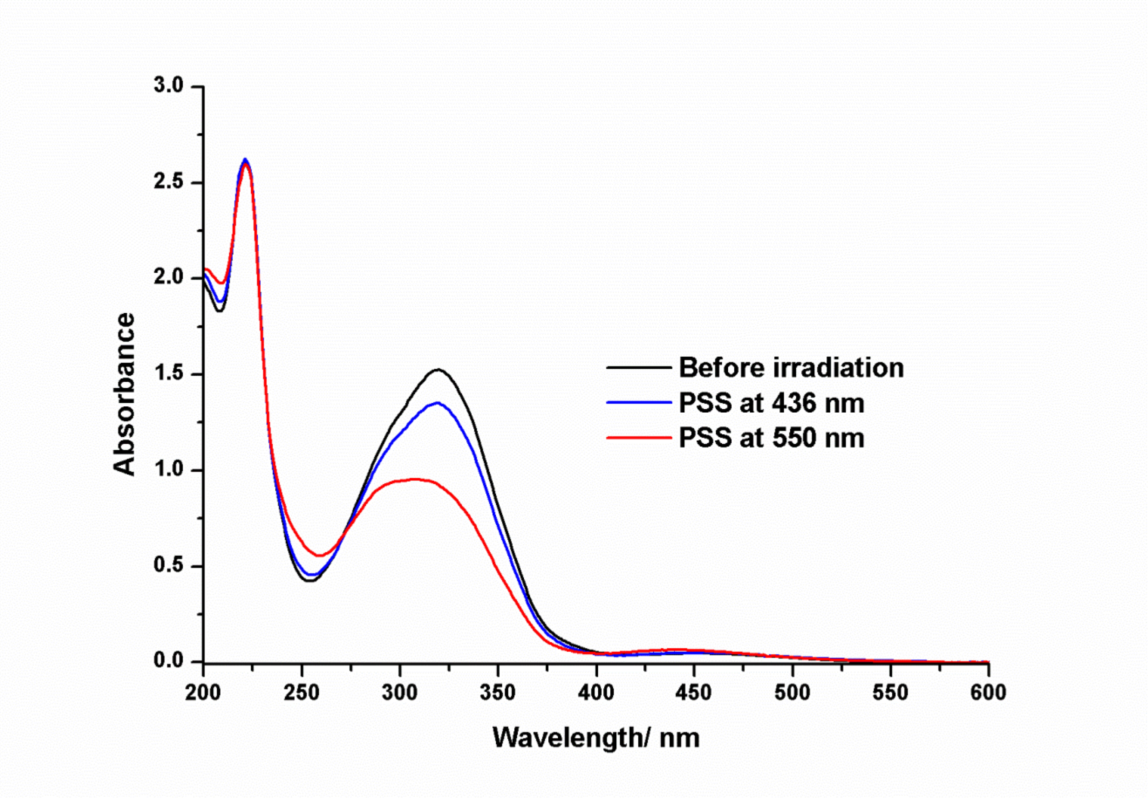

Figure S5. Visible light switching of **3** in MeCN at room temperature.

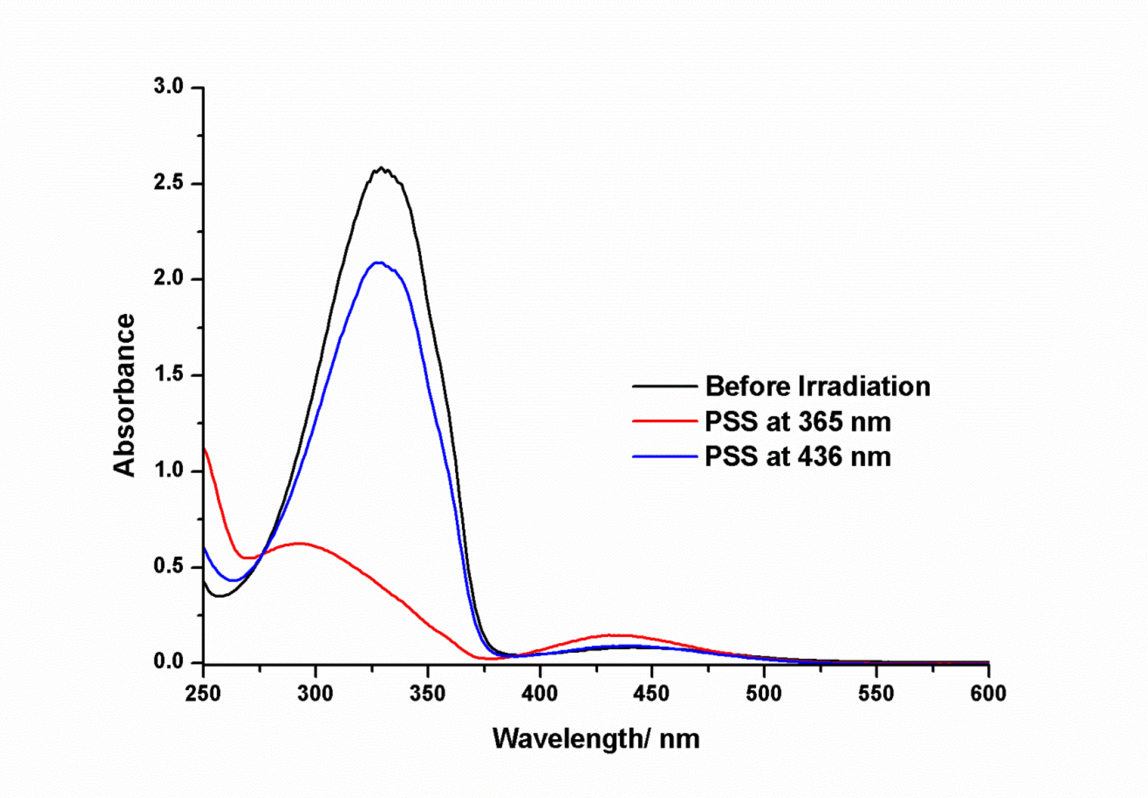

Figure S6. Absorption spectra of compound **1** in MeCN (5.1×10^{-4} M) before and after irradiation at 365 and 436 nm.

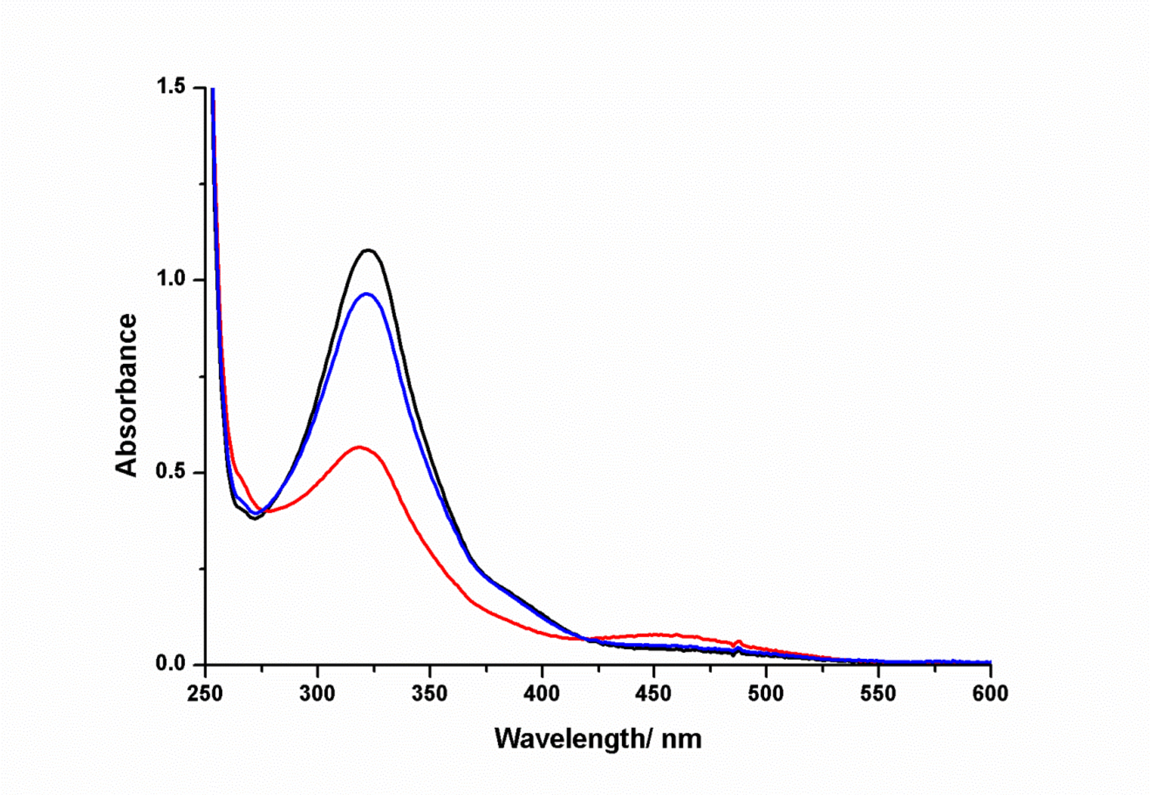

Figure S7. Absorption spectra of compound **2** in MeCN (3.69×10^{-4} M) before (black line) and after irradiation at 365 (red line) and 436 (blue line) nm.

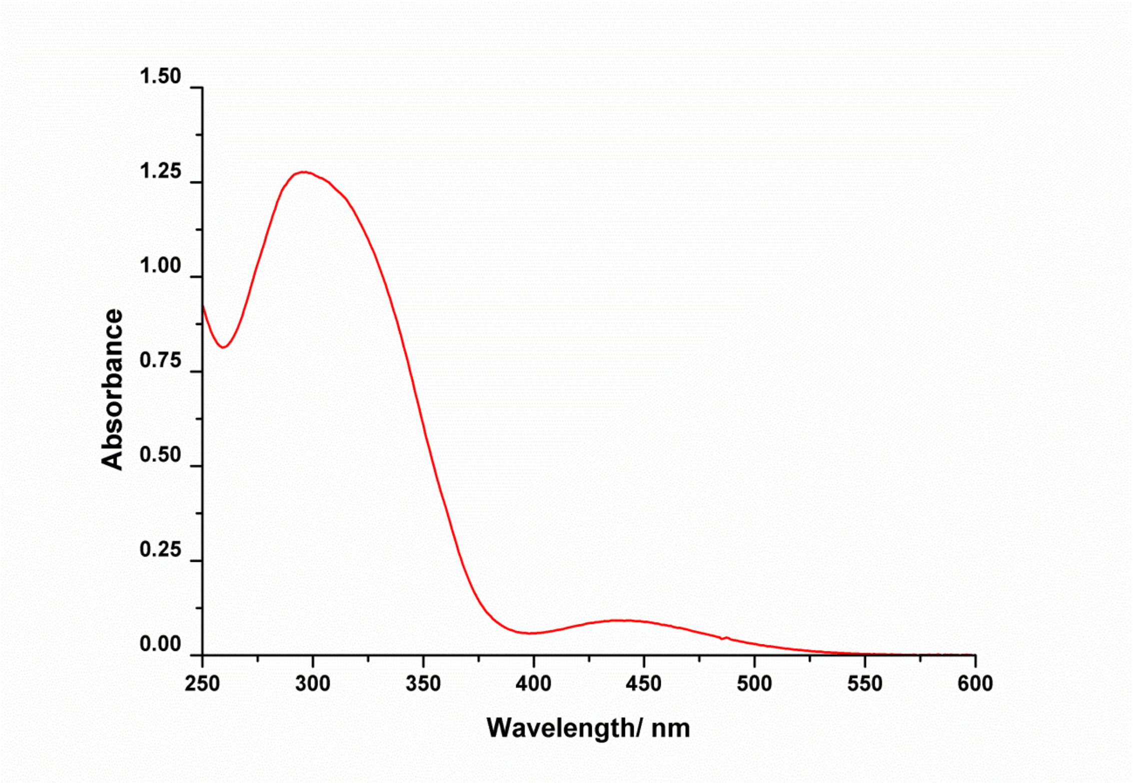

Figure S8. Absorption spectrum of *EZ*-$\mathbf{3_A}$ in MeCN after HPLC separation. An identical spectrum was obtained for *EZ*-$\mathbf{3_B}$.

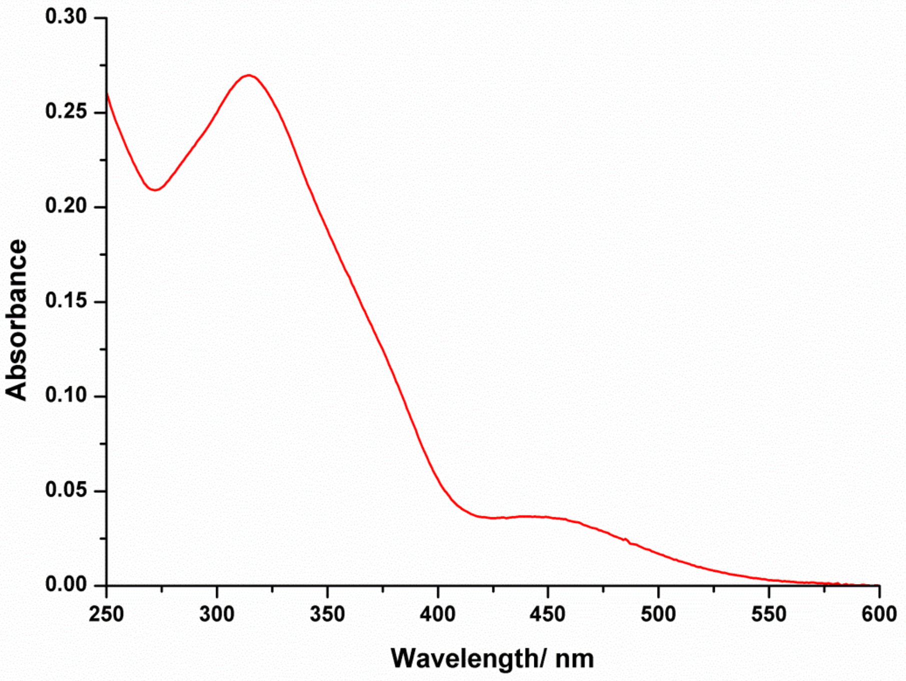

Figure S9. Absorption spectrum of *EZ*-$\mathbf{2}_\mathbf{A}$ in MeCN after HPLC separation. An identical spectrum was obtained for *EZ*-$\mathbf{2}_\mathbf{B}$.

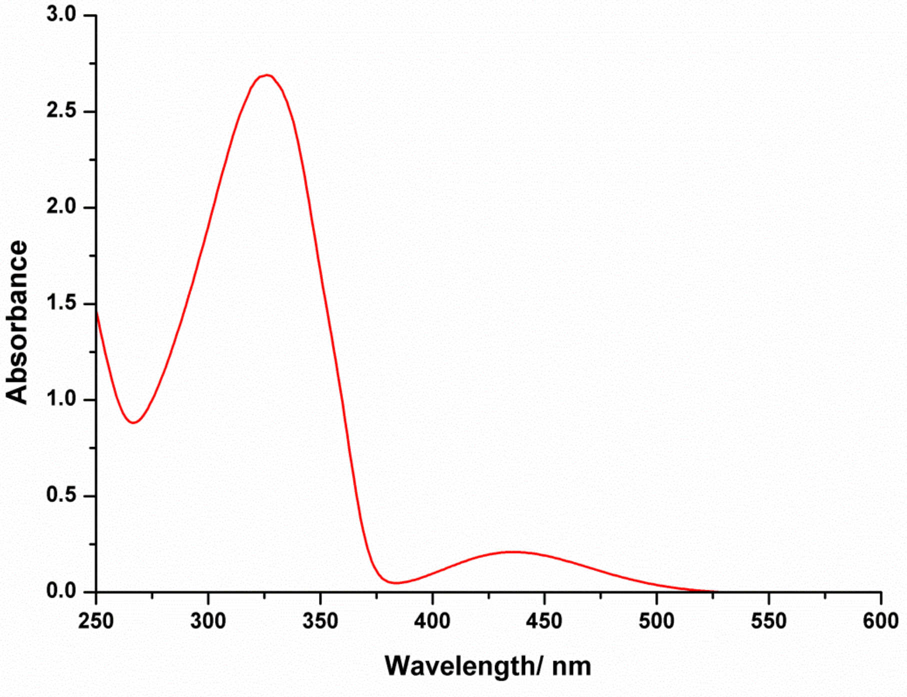

Figure S10. Absorption spectrum of *EZ*-$\mathbf{1}_\mathbf{A}$ in MeCN after HPLC separation. An identical spectrum was obtained for *EZ*-$\mathbf{1}_\mathbf{B}$.

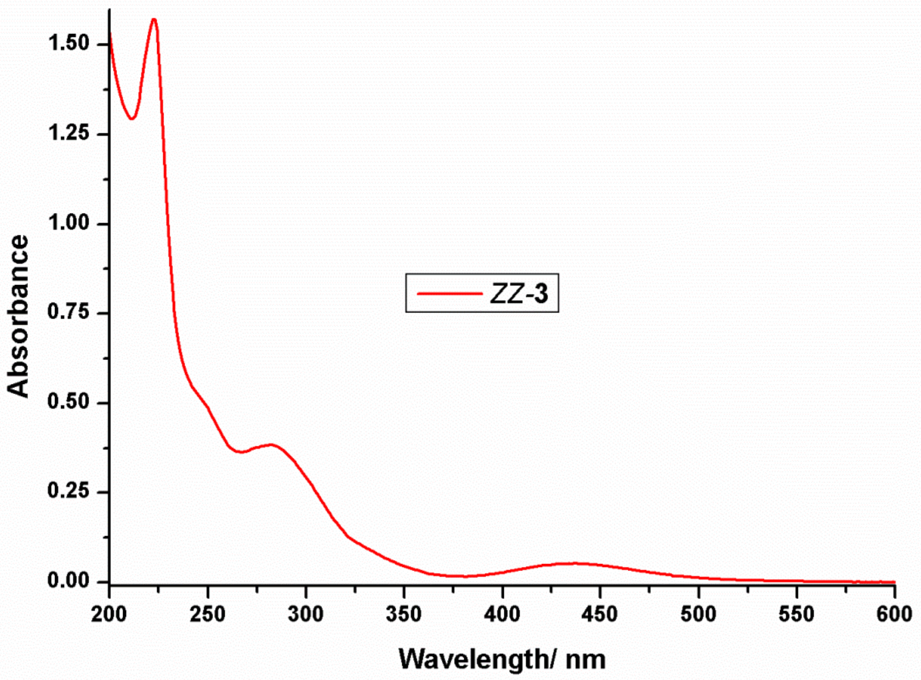

Figure S11. Absorption spectrum of *ZZ*-**3** in MeCN after HPLC separation.

5. HPLC Chromatogram

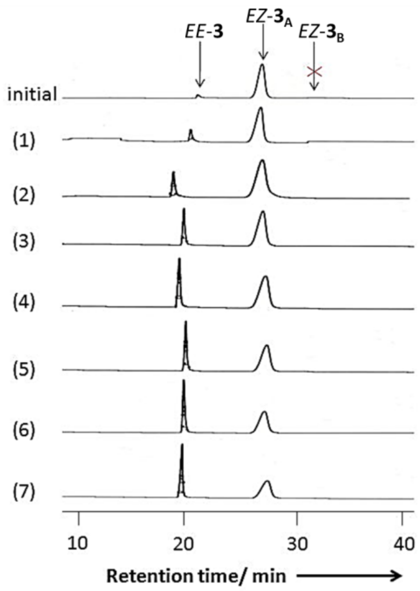

Figure S12. HPLC profile revealing the absence of racemization during the thermal back-isomerization of *EZ*-$\mathbf{3_A}$, the first-eluted enantiomer of *EZ*-**3**, in the dark at room temperature over one week.

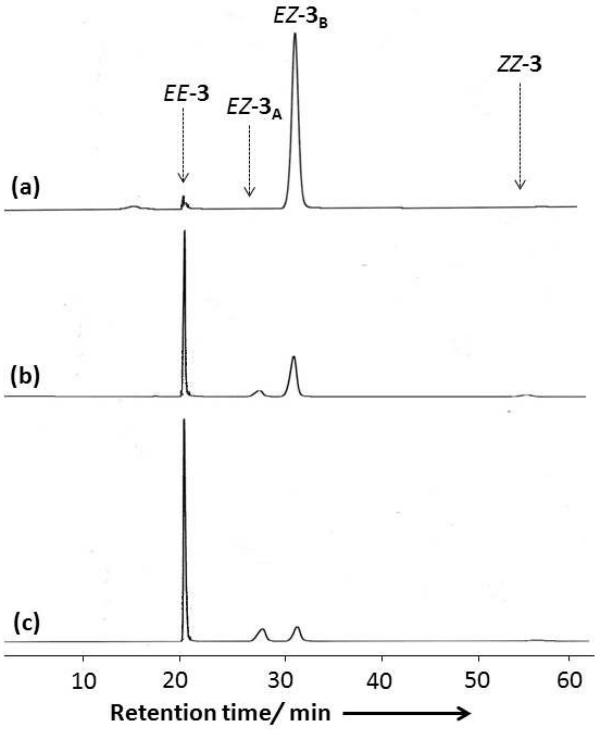

Figure S13. HPLC chromatograms of *EZ*-$\mathbf{3_B}$, the second-eluted enantiomer of *EZ*-**3**: (a) before irradiation, (b) after irradiation for 10 s at 436 nm, and (c) the PSS at 436 nm.

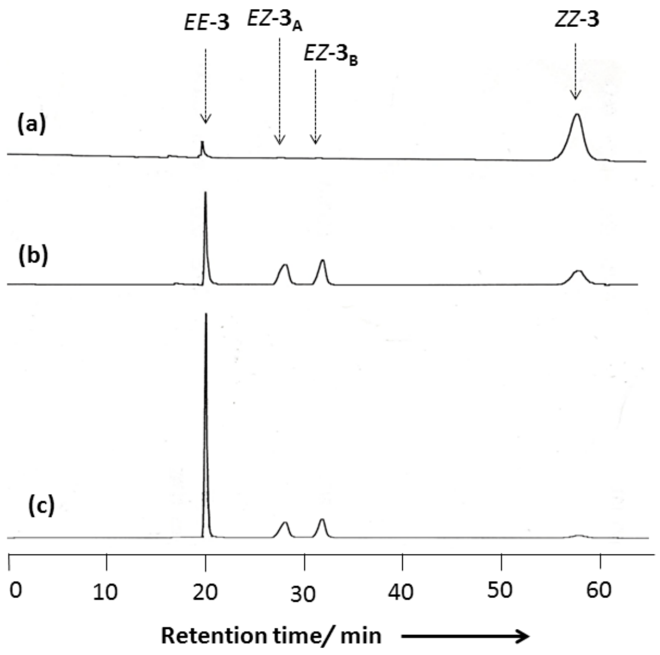

Figure S14. HPLC chromatograms revealing the *ZZ*-to-*EE* photoisomerization pathway of compound **3** via the *EZ* intermediate: (a) before irradiation, (b) after irradiation for 10 s at 436 nm, and (c) the PSS at 436 nm.

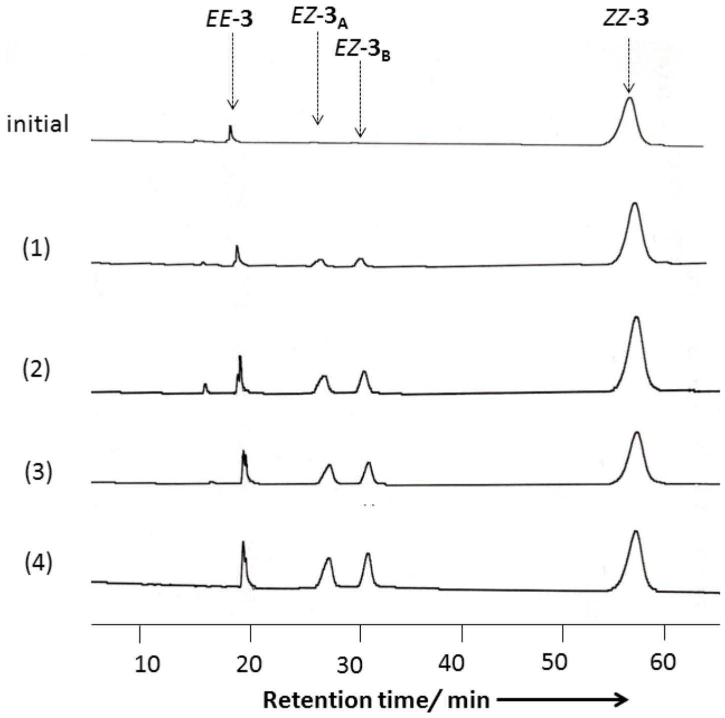

Figure S15. HPLC chromatograms revealing the *ZZ*-to-*EE* thermal isomerization of compound **3** via the *EZ* intermediate, in the dark at room temperature.

6. CD Spectra

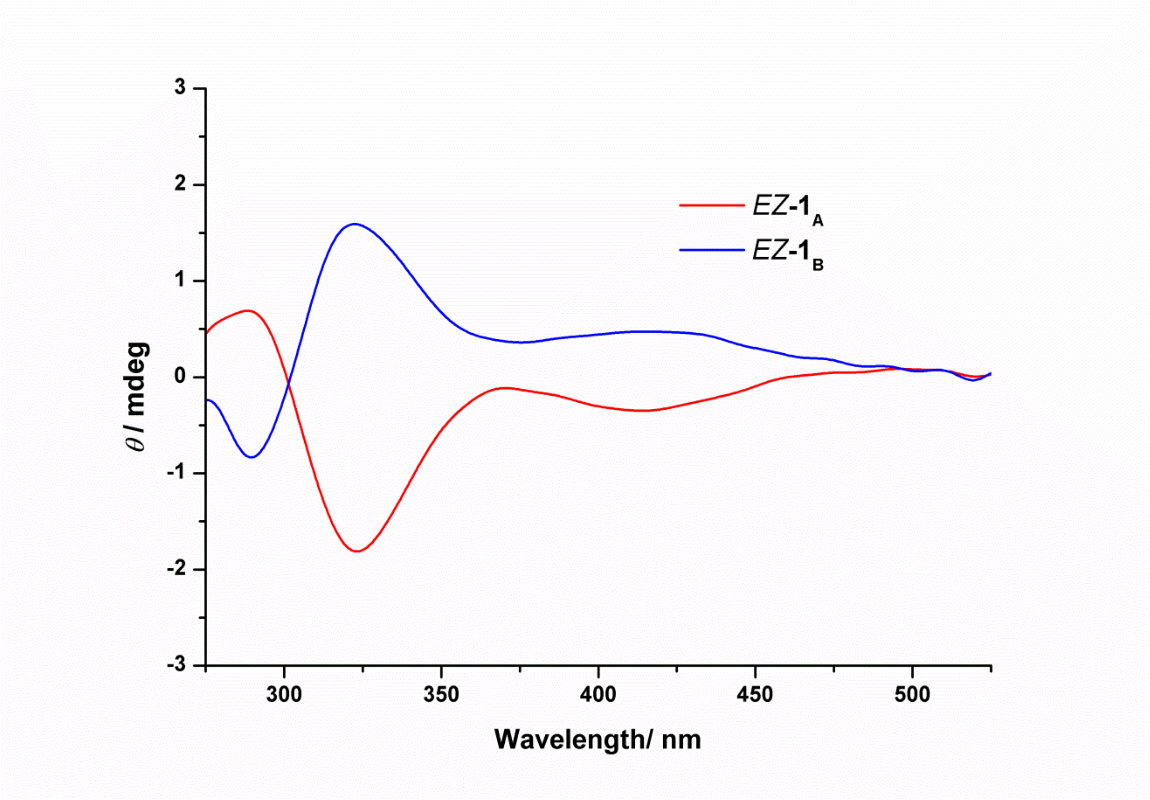

Figure S16. CD spectra of *EZ*-**1** in MeCN; (red line) first-eluted enantiomer, *EZ*-$\mathbf{1_A}$; (blue line) second-eluted enantiomer, *EZ*-$\mathbf{1_B}$. Concentration of each solution: 1.01×10^{-4} M.

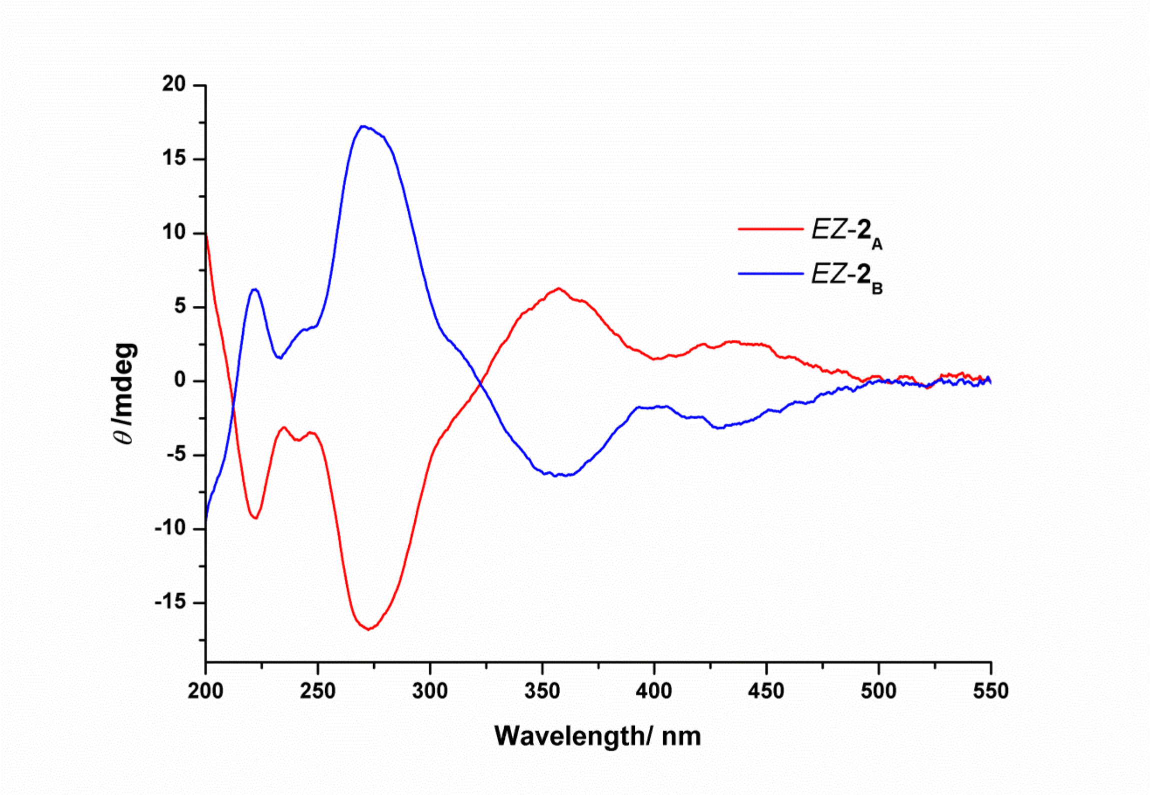

Figure S17. CD spectra of *EZ*-**2** in MeCN; (red line) first-eluted enantiomer, *EZ*-$\mathbf{2_A}$; (blue line) second-eluted enantiomer, *EZ*-$\mathbf{2_B}$. Concentration of each solution: 1.94×10^{-4} M.

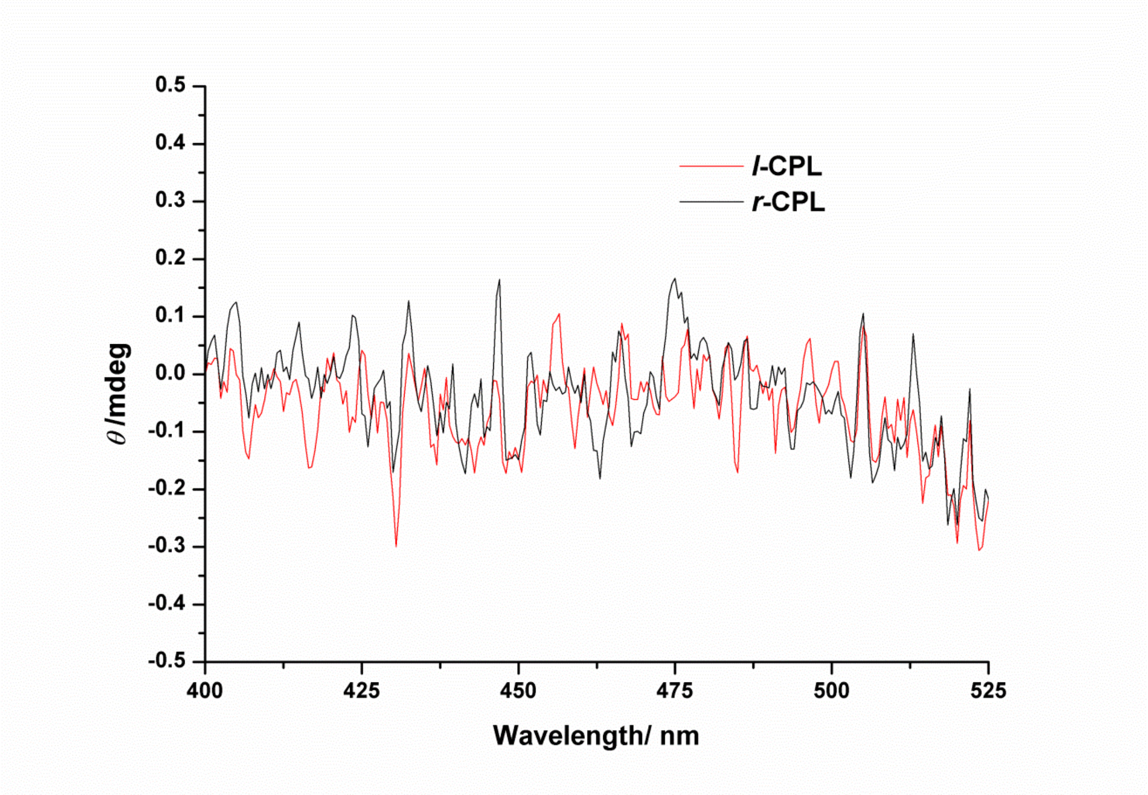

Figure S18. CD spectra of a solution of **1** (1.42×10^{-3} M) in MeCN after irradiation with *r*-CPL (black line) and *l*-CPL (red line), revealing no induced CD at PSS_{436}.

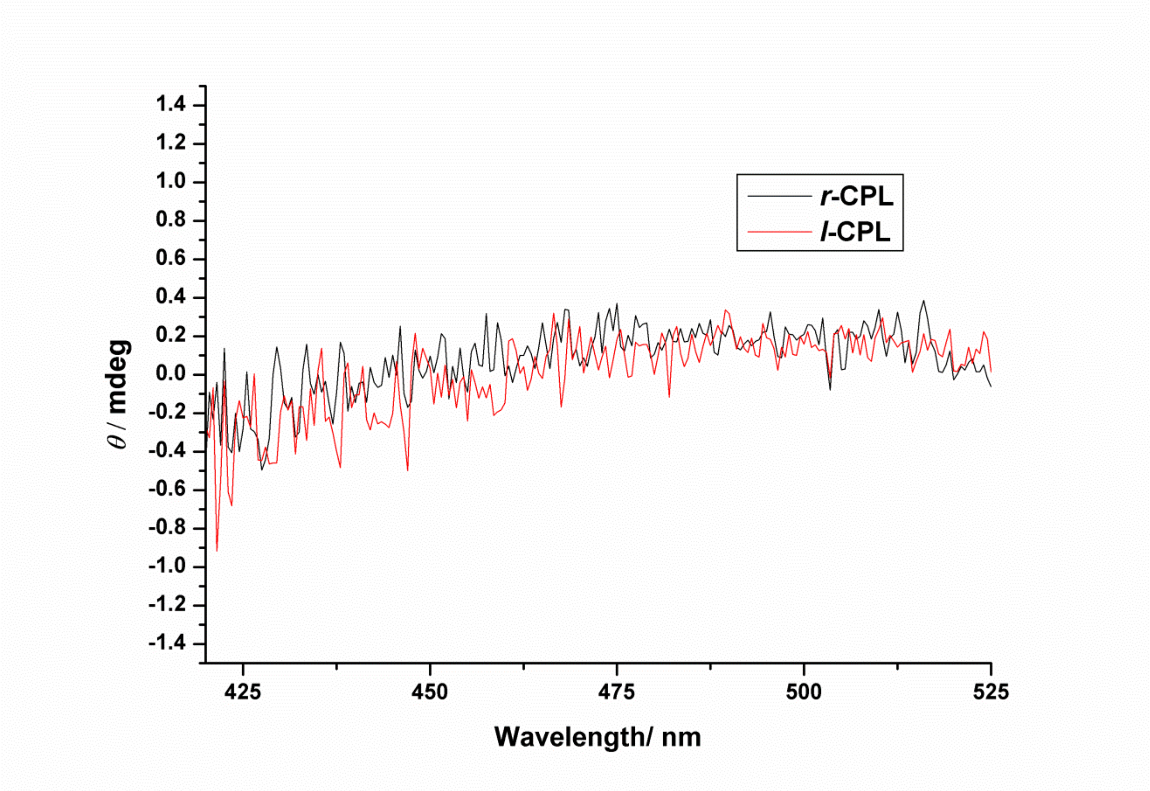

Figure S19. CD spectra of a solution of **2** (1.5×10^{-3} M) in MeCN after irradiation with *r*-CPL (black line) and *l*-CPL (red line), revealing no induced CD at PSS_{436}.

7. Kinetic studies

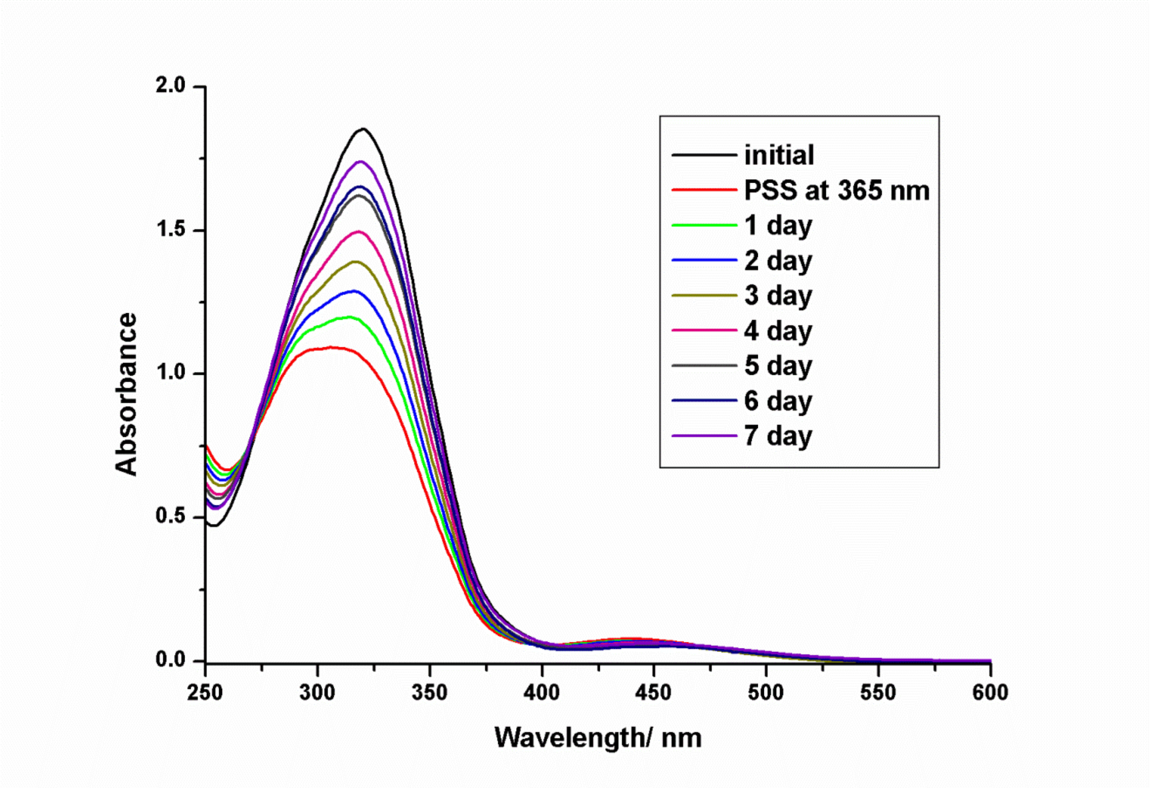

Figure S20. UV spectra of a solution of **3** (1.5×10^{-3} M) in MeCN during thermal back-isomerization after $PSS_{365\ nm}$, at 30 °C in the dark for 7 days.

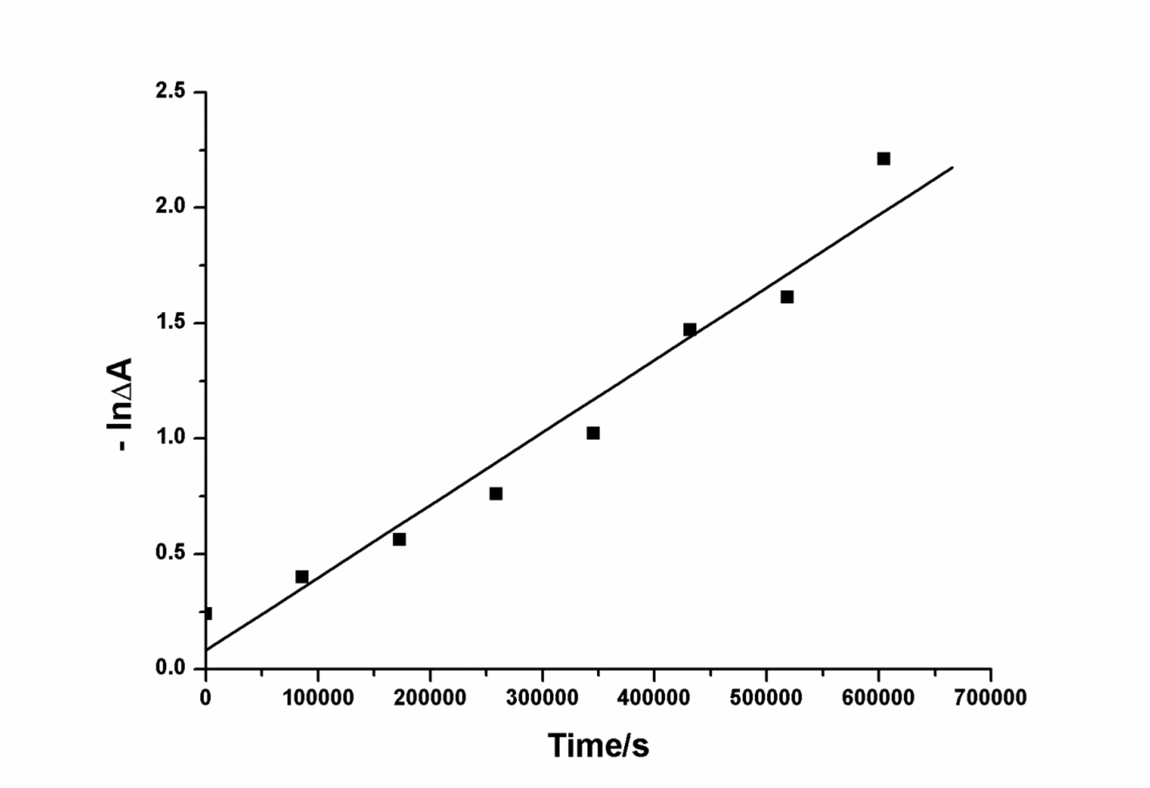

Figure S21. Linear relationship between the logarithm of the change in absorbance ($\Delta A = A_I - A_t$, determined from the absorbance at 320 nm in the UV spectra, where A_I is the initial absorbance before irradiation and A_t is the absorbance at time t after $PSS_{366\ nm}$) and time for **3** after $PSS_{365\ nm}$ at 30 °C.

8. Estimation of the enantiomeric excess after CPL irradiation

The enantiomeric excess in the photoresolution process is estimated by the following calculation,

The photochemical rate equations in a 1cm cell are

$$\frac{d\,[R-EZ]}{dt} = 1000I_0\,\{(1\text{-}10^{-A})/A\}\{(\varepsilon_{EE}\ \phi_{EE\rightarrow EZ}[EE] + \varepsilon_{ZZ}\phi_{ZZ\rightarrow EZ}[ZZ]) - (\varepsilon_{R\text{-}EZ}\phi_{R\text{-}EZ\rightarrow EE}[R\text{-}EZ] + \varepsilon_{R\text{-}EZ}\phi_{R\text{-}EZ\rightarrow ZZ}[R\text{-}EZ])\} \qquad (1)$$

$$\frac{d\,[S-EZ]}{dt} = 1000I_0\,\{(1\text{-}10^{-A})/A\}\{(\varepsilon_{EE}\ \phi_{EE\rightarrow EZ}[EE] + \varepsilon_{ZZ}\phi_{ZZ\rightarrow EZ}[ZZ]) - (\varepsilon_{S\text{-}EZ}\phi_{S\text{-}EZ\rightarrow EE}[S\text{-}EZ] + \varepsilon_{S\text{-}EZ}\phi_{S\text{-}EZ\rightarrow ZZ}[S\text{-}EZ])\} \qquad (2)$$

where $[R\text{-}EZ]$, $[S\text{-}EZ]$, $[EE]$, $[ZZ]$ and $\varepsilon_{R\text{-}EZ}$, $\varepsilon_{S\text{-}EZ}$, ε_{EE}, ε_{ZZ} are concentrations and molar extinction coefficients of *R-EZ*, *S-EZ*, *EE*, *ZZ* isomers, respectively, and $\phi_{EE\rightarrow EZ}$, $\phi_{ZZ\rightarrow EZ}$, $\phi_{EZ\rightarrow EE}$, $\phi_{EZ\rightarrow ZZ}$ represents the quantum yields of photochemical isomerization from $EE\rightarrow EZ$, $ZZ\rightarrow EZ$, $EZ\rightarrow EE$, $EZ\rightarrow ZZ$, respectively.

At the photostationary state,

$$\frac{d\,[R-EZ]}{dt} = \frac{d\,[S-EZ]}{dt} = 0 \qquad (3)$$

Then,

$$\varepsilon_{EE}\ \phi_{EE\rightarrow EZ}[EE] + \varepsilon_{ZZ}\phi_{ZZ\rightarrow EZ}[ZZ] = \varepsilon_{R\text{-}EZ}\ \phi_{R\text{-}EZ\rightarrow EE}[R\text{-}EZ] + \varepsilon_{R\text{-}EZ}\phi_{R\text{-}EZ\rightarrow ZZ}[R\text{-}EZ] \qquad (4)$$

$$\varepsilon_{EE}\ \phi_{EE\rightarrow EZ}[EE] + \varepsilon_{ZZ}\phi_{ZZ\rightarrow EZ}[ZZ] = \varepsilon_{S\text{-}EZ}\ \phi_{S\text{-}EZ\rightarrow EE}[S\text{-}EZ] + \varepsilon_{S\text{-}EZ}\phi_{S\text{-}EZ\rightarrow ZZ}[S\text{-}EZ] \qquad (5)$$

Equating 4 and 5,

$$\varepsilon_{R\text{-}EZ}\phi_{R\text{-}EZ\rightarrow EE}[R\text{-}EZ] + \varepsilon_{R\text{-}EZ}\phi_{R\text{-}EZ\rightarrow ZZ}[R\text{-}EZ] = \varepsilon_{S\text{-}EZ}\phi_{S\text{-}EZ\rightarrow EE}[S\text{-}EZ] + \varepsilon_{S\text{-}EZ}\phi_{S\text{-}EZ\rightarrow ZZ}[S\text{-}EZ] \qquad (6)$$

$$\varepsilon_{R\text{-}EZ}[R\text{-}EZ]\,(\phi_{R\text{-}EZ\rightarrow EE} + \phi_{R\text{-}EZ\rightarrow ZZ}) = \varepsilon_{S\text{-}EZ}[S\text{-}EZ]\,(\phi_{S\text{-}EZ\rightarrow EE} + \phi_{S\text{-}EZ\rightarrow ZZ}) \qquad (7)$$

Since *R-EZ* and *S-EZ* enantiomers are chemically same, the interconversion quantum yields must be identical for symmetry reasons.

Therefore equation 7 can be written as

$$\varepsilon_{R\text{-}EZ}[R\text{-}EZ] = \varepsilon_{S\text{-}EZ}[S\text{-}EZ] \qquad (8)$$

This leads, as $\varepsilon_{R\text{-}EZ} = \varepsilon_{EZ} - (\Delta\varepsilon_{EZ}/2)$ and $\varepsilon_{S\text{-}EZ} = \varepsilon_{EZ} + (\Delta\varepsilon_{EZ}/2)$, to

$$\{\varepsilon_{EZ} - (\Delta\varepsilon_{EZ}/2)\}\,[R\text{-}EZ] = \{\varepsilon_{EZ} + (\Delta\varepsilon_{EZ}/2)\}[S\text{-}EZ] \qquad (9)$$

Then, equation 9 can be deduced to,

$$\varepsilon_{EZ}\{[R\text{-}EZ] - [S\text{-}EZ]\} = (\Delta\varepsilon_{EZ}/2)\,\{[R\text{-}EZ] + [S\text{-}EZ]\} \qquad (10)$$

$$([R\text{-}EZ] - [S\text{-}EZ])\,/\,([R\text{-}EZ] + [S\text{-}EZ]) = \Delta\varepsilon_{EZ}/2\varepsilon_{EZ} \qquad (11)$$

Kuhn anisotropy factor, g is defined as,

$$g = \Delta\varepsilon/\varepsilon \qquad (12)$$

from the equations 11 and 12, equation 13 can be obtained,

$$([R\text{-}EZ] - [S\text{-}EZ])\,/\,([R\text{-}EZ] + [S\text{-}EZ]) = g_{EZ}/2 \qquad (13)$$

Table S1. Crystallographic data of **3**.

	**3**
Formula	$C_{28}H_{20}N_4$
Formula weight	412.49
Crystal system	Triclinic
Space group	*P*1–
a / Å	7.8497(5)
b / Å	10.7861(8)
c / Å	14.7876(10)
α / °	109.560(3)
β / °	91.8983(18)
γ / °	111.312(3)
V / Å^3	1081.88(13)
Z	2
Crystal size / mm	0.50 × 0.30 × 0.30
T / K	173
D_c / g cm^{-3}	1.266
F_{000}	432.00
λ / Å	0.71075
μ(Mo-Kα) / cm^{-1}	0.763
Data measured	10582
Data unique	4868
R_{int}	0.0242
No. of observations	4868
No. of variables	289
R_1 ($I > 2.00\sigma(I)$)^a	0.0503
R (all reflections)^a	0.0597
wR_2 (all reflections)^b	0.1454
GOF	1.065
CCDC number	1003754

^{a} $R_1 = R = \Sigma \,||F_o| - |F_c|| / \Sigma |F_o|$. ^{b} $wR_2 = [\Sigma\, w(F_o^2 - F_c^2)^2 / \Sigma\, w(F_o^2)^2]^{1/2}$.
